# Multivariate analysis and genetic dissection of staygreen and stem reserve mobilisation under combined drought and heat stress in wheat (*Triticum aestivum* L.)

**DOI:** 10.3389/fgene.2023.1242048

**Published:** 2023-08-29

**Authors:** Sukumar Taria, Ajay Arora, Hari Krishna, Karthik Kumar Manjunath, Shashi Meena, Sudhir Kumar, Biswabiplab Singh, Pavithra Krishna, Animireddy China Malakondaiah, Ritwika Das, Badre Alam, Sushil Kumar, Pradeep Kumar Singh

**Affiliations:** ^1^ Division of Plant Physiology, ICAR-Indian Agricultural Research Institute, New Delhi, India; ^2^ ICAR-Central Agroforestry Research Institute, Jhansi, Uttar Pradesh, India; ^3^ Division of Genetics, ICAR-Indian Agricultural Research Institute, New Delhi, India; ^4^ Division of Agricultural Bioinformatics, ICAR-Indian Agricultural Statistics Research Institute, New Delhi, India

**Keywords:** wheat, staygreen, stem reserve mobilisation, QTLs, drought, heat

## Abstract

**Introduction**: Abiotic stresses significantly reduce crop yield by adversely affecting many physio-biochemical processes. Several physiological traits have been targeted and improved for yield enhancement in limiting environmental conditions. Amongst them, staygreen and stem reserve mobilisation are two important mutually exclusive traits contributing to grain filling under drought and heat stress in wheat. Henceforth, the present study was carried out to identify the QTLs governing these traits and to identify the superiors’ lines through multi-trait genotype-ideotype distance index (MGIDI)

**Methods**: A mapping population consisting of 166 recombinant inbred lines (RILs) developed from a cross between HD3086 and HI1500 was utilized in this study. The experiment was laid down in alpha lattice design in four environmental conditions viz. Control, drought, heat and combined stress (heat and drought). Genotyping of parents and RILs was carried out with 35 K Axiom^®^ array (Wheat breeder array).

**Results and Discussion**: Medium to high heritability with a moderate to high correlation between traits was observed. Principal component analysis (PCA) was performed to derive latent variables in the original set of traits and the relationship of these traits with latent variables.From this study, 14 QTLs were identified, out of which 11, 2, and 1 for soil plant analysis development (SPAD) value, leaf senescence rate (LSR), and stem reserve mobilisation efficiency (SRE) respectively. Quantitative trait loci (QTLs) for SPAD value harbored various genes like Dirigent protein 6-like, Protein FATTY ACID EXPORT 3, glucan synthase-3 and Ubiquitin carboxyl-terminal hydrolase, whereas QTLs for LSR were found to contain various genes like aspartyl protease family protein, potassium transporter, inositol-tetrakisphosphate 1-kinase, and DNA polymerase epsilon subunit D-like. Furthermore, the chromosomal region for SRE was found to be associated with serine-threonine protein kinase. Serine-threonine protein kinases are involved in many signaling networks such as ABA mediated ROS signaling and acclimation to environmental stimuli. After the validation of QTLs in multilocation trials, these QTLs can be used for marker-assisted selection (MAS) in breeding programs.

## Introduction

Wheat (*Triticum aestivum* L.) is a cool-season crop grown worldwide in many different agroecological conditions and cropping systems (Talaat, 2021). Wheat production is simultaneously challenged by drought and heat waves (El-Habti et al., 2020). It is reported that drought and heat stress are two significant abiotic stresses (Bhunia et al., 2020; Valipour et al., 2021) that cause substantial yield losses in wheat, up to 86% and 69%, respectively, hence limiting wheat’s productivity (Prasad et al., 2011; Yang et al., 2021). Moreover, the effect of combined stress (drought and heat) is more detrimental than individual stress (Mittler, 2006). It is predicted that there is a chance of crossing the global warming level of 1.5°C in the next decades (IPCC, 2021). In combination drought and heat stress, significantly reduced the photosynthetic system, gaseous exchange and plant water relation (Shah and Paulsen, 2003). Heat stress response was shown to be modulated by enhanced CO_2_ levels through the expression of isoflavone reductase like (IRL) gene, which was associated with photosynthetic capacity of leaves, antioxidant capacity, and hormonal (cytokinin) regulation (Shokat et al., 2021). It has been demonstrated that the activity of monodehydroascorbate reductase (MDHAR), leaf aldolase and cell wall peroxidase (cwPOX) were significantly correlated with thousand kernel weight and grain numbers in wheat under well-watered and drought conditions respectively (Shokat et al., 2020). Furthermore, it has been also stated that enhanced CO_2_ levels alleviated the negative effect of drought stress by modulating carbohydrate metabolic enzyme activity and antioxidant potential in wheat (Ulfat et al., 2021). Therefore, these physio-biochemical and molecular markers can be used for the selection of climate resilient wheat genotypes for upcoming food demand. Moreover, drought and heat tolerance can be improved in bread wheat by exploiting pre-breeding traits and its genetic diversity at reproductive stage (Shokat et al., 2023). To meet the global food demand for a growing population by 2050, it is important to enhance the performance of wheat under stress conditions. Traits related to developmental phases, physiological and hydraulic traits can be targeted to improve the tolerance to combined drought and heat stress in wheat (Tricker et al., 2018).

Amongst the physiological traits, staygreen (SG) and stem reserve mobilisation (SRM) are the traits which can contribute to yield enhancement under drought and heat stress (Ram et al., 2018; Kumar et al., 2021a; Gurumurthy et al., 2023). Staygreen is a trait in which leaves retain greenness from flowering to physiological maturity instead of senescing and it is a well-known trait for grain filling (Zhang et al., 2019). Senescence is a genetically programmed and environmentally influenced process resulting in the destruction of chlorophyll and the remobilization of nutrients to younger or reproductive parts of plants (Vijayalakshmi et al., 2010). Furthermore, grain filling in cereals depend on fixed carbon from two sources-current leaf photosynthesis and remobilization of stored carbohydrates in the stem during pre- and post-anthesis periods (Yang and Zhang, 2006; Joudi et al., 2012). It is reported that cultivars having a higher capacity of SRM for grain filling had higher senescence rates under normal and stress conditions in wheat (Blum et al., 1994; Fokar et al., 1998). The plant hormone abscisic acid (ABA) has been reported to be involved in the enhanced remobilisation of assimilates from vegetative tissue to reproductive organs during the period of water stress (Travaglia et al., 2012). It has been reported that the application of ABA increased the remobilisation of carbon from photosynthetic tissue to grain in wheat and rice during water stress respectively (Yang et al., 2001a, b). Moreover, it is established that SG as a source of current assimilation and SRM at another end for transporting storage reserve to grain may be mutually exclusive (Blum, 1988). Therefore, attention is needed to develop wheat varieties with the potential to maintain greenness for longer periods and higher mobilisation of water-soluble carbohydrates (WSC) to grain for enhancing crop yield. The genetic basis of staygreen has been reported in wheat (Simon, 1999) and an additive gene effect was also reported (Joshi et al., 2007). QTLs related to staygreen have been reported by several workers (Kumar et al., 2012; Yang et al., 2016). Mobilisation related traits have also been reported by previous workers such as SRM (Salem et al., 2007; Yang et al., 2007), and water-soluble carbohydrates (Bennett et al., 2012) using different mapping populations. However, only a few QTLs have been identified with high phenotypic variance (Manjunath et al., 2023). Furthermore, multivariate data is very common in biological experiments. The selection of superior genotypes or donors’ parents is critical due to the presence of multicollinearity between traits of interest (Olivoto and Nardino, 2021). Though Smith-Hazel (SH) index is widely used for genotypes selection, there is much evidence that it is not being used either in early breeding trials (Bhering et al., 2012) or in advanced stages of breeding programs (Jahufer and Casler, 2015). Currently, the multi-trait genotype–ideotype distance index (MGIDI) is used for genotype selection in multi-environment trials based on desired idiotypes free from weighting coefficients and multicollinearity issues (Olivoto and Nardino, 2021).

Staygreen potential and higher SRM were mutually exclusive (Blum, 1998). We hypothesized that it would be possible to enhance grain filling by combining these traits into a single genotype/line. To achieve this objective, parents (HD3086 and HI1500) having contrasting traits (staygreen and stem reserve mobilisation) were selected and crossed to develop recombinant inbred lines (RILs). Thereafter, phenotyping was done for both these traits (SPAD, LSR, SRM) under control, drought, heat, and combined stress conditions. Inclusive composite interval mapping (ICIM) approach was followed to identify QTLs for staygreen (SPAD value and LSR) and stem reserve mobilisation efficiency. Bioinformatics approaches were used to select putative candidate genes for staygreen and SRM. To select the lines with both these traits contributing to the grain filling, multi-trait genotype ideotypes distance index (MGIDI), a model widely applied in multi-environment data analysis was used.

## Materials and methods

This study was carried out using a mapping population consisting of 166 recombinant inbred lines (RILs) developed by the crossing of HD3086 and HI1500. HD3086 is a high-yielding hexaploid wheat variety suitable for irrigated conditions (timely sown), whereas HI1500 is suitable for restricted irrigated conditions attributed due to its drought and heat tolerance conditions (Singh et al., 2014). The RILs along with their parents were evaluated under timely sowing irrigated (control) and restricted irrigation (drought) along with late sown with irrigation (heat stress) and less irrigation (combined heat and drought stress) during rabi season 2021–22. The experiment was conducted using an alpha lattice design with two replications. Each genotype was sown in 3 rows of 1 m each with a 22.5 cm distance between rows and a 10 cm distance between plants. Proper agronomic practices were followed for uniform plant establishment under field conditions. The selection of superior lines has been carried out using 220 RILs of the same parents.

The minimum and maximum temperatures (°C), as well as precipitation (mm) during the wheat crop development season (2021–22), are depicted in Supplementary Figure S1. The mean plant available water content in the root zone at the anthesis stage was 25.14% for control, 18.47% for drought, 23.82% for heat, and 14.69% for the combined stress conditions.

### Phenotyping for staygreen traits and stem reserve mobilisation efficiency (SRE)

To evaluate the staygreen capacity of the population, SPAD chlorophyll content and leaf senescence rate was measured. SPAD chlorophyll content was recorded at anthesis, 10 days after anthesis (DAA), and 20 DAA under timely sown conditions (control and drought). Under heat stress, SPAD values were recorded at anthesis, 10 DAA, 15 DAA, and 20 DAA, whereas under combined stress SPAD chlorophyll content was recorded at anthesis, 5 DAA, and 10 DAA. The leaf turned to complete yellow following anthesis was scored 0 for SPAD value.

Phenotyping for leaf senescence was done visually and scored using a scale from 0 to 10, dividing the percentage of the estimated dead area by time duration in days as per the method described by Lu et al. (2011) and Shivramakrishnan et al. (2016). A rating of 10 indicated essentially no leaf death, 5-6 indicated approximately 50% mature leaf area dead, while 0 indicate 100% leaf senescence. 10 = no leaf dead area; 9 = 10% dead area; 8 = 20% dead area; 7 = 30% dead area; 6 = 40% dead area; 5 = 50% dead area; 4 = 60% dead area; 3 = 70% dead area; 2 = 80% dead area; 1 = 90% dead area and 0 = 100% dead area. LSR was scored from 15 days anthesis stage at every 2 days interval in control and drought stress, whereas LSR was scored 10 DAA in heat and combined stress conditions. The average LSR was calculated using the formula.

$$LSR=\frac{Initial\;score-Final\;score}{No.\;of\;scoring\;days}$$

Phenotyping for stem reserve mobilisation was carried out as per the methods described by Ehdaie et al. (2006). The main shoots of wheat plants (6 plants) were tagged in the field having the same date of anthesis. Defoliation of all leaves (3 plants) including flag leaves was done 12 DAA and the main shoots were chopped and kept for over-drying. Leaves of the remaining 3 plants were removed and kept in the field up to physiological maturity. SRE was calculated using the following formulae-

$$Stem\;reserve\;mobilisation\;efficiency=\frac{Stem\;weight\;at\;12\;DAA-Stem\;weight\;at\;physiological\;maturity}{Stem\;weight\;at\;12\;DAA}\times 100$$

### Statistical analysis

For the removal of the outliers, the interquartile range (IQR) method was followed. For the calculation of lower and upper values, the following formula is used-

$$Lower=Quantile\left(25^{th}\right)-1.5*IQR$$

$$Upper=Quantile\left(75^{th}\right)+1.5*IQR$$

Two-way ANOVA has been conducted using Metan package with anova_joint functions to visualise the interaction between genotypes x treatments and one-way analysis of variance was carried out using PBIB function. Heritability (h^2^), genetic coefficients of variance (GCV) and least significance difference (LSD) value were calculated using the MetaRv6.0 (Multi Environment Trial Analysis with R) software. Pearson’s correlation was performed to illustrate the dependency between the variables using the psych package with the following mathematical equation-

$$r=\frac{\sum \left(x_{i}-\bar{x}\right)\;\left(y_{i}-\bar{y}\right)}{\sqrt{\sum {\left(x_{i}-\bar{x}\right)}^{2}\sum {\left(y_{i}-\bar{y}\right)}^{2}}}$$

Where.r = correlation co-efficient. x_i_ = value of x variables in the sample x. $\bar{x}$ = mean of the values of the x variables, y_i_ = value of x variables in the sample y. $\bar{y}$ = mean of the values of the y variables

Principal component analysis was performed to reduce the variable redundancy in traits as well as to reduce multicollinearity. Principal components were extracted using FactomineR package and enhanced visualization was carried out using factoextra package. PCA was conducted by normalization of data set by standardization followed by computing covariance matrix. The covariance matrix of two-dimensional data is given as follows -

$$Covariance\;matrix=\left[\begin{array}{ccc} COV\left(X,X\right) & \cdots & COV\;\left(X,Y\right)\\ \vdots & \ddots & \vdots \\ COV\left(Y,X\right), & \cdots & COV\;\left(Y,Y\right) \end{array}\right]$$

From phenotypic data, best linear unbiased predictors (BLUPs) were calculated for the individual conditions and combined across all production conditions and environments for further QTL mapping. BLUP values were calculated using META-R, where it calculate BLUPs for all traits when genotypes are considered with random effects and the BLUP for each genotype is the grand mean added to the estimated random effect from each genotype.

### Selection of superior RILs

Selection of superior lines in the RILs population across the multi-environmental trials was carried out by computing multi-trait genotype ideotypes distance index (MGIDI) based on ideotypes input (Olivoto and Nardino, 2021). MGIDI is calculated as follows-

$$MGIDI=\sqrt{\sum_{j=1}^{f}{\left(F_{ij}-F_{j}\right)}^{2}}$$

Where MGIDI is the multi-trait genotype ideotypes distance index, F_ij_ is the score of the i^th^ genotype in the j^th^ factor (i = 1, 2, … , g; j = 1, 2, … , f), being g and f the number of genotypes and factors, respectively, and F_j_ is the j^th^ score of the ideotype. For the ideotype plan, LSR and SSI were given as lower values, and the rest of the traits were given as higher values. All statistical analysis has been performed using R programming version 4.2.2.

### DNA extraction and genotyping

The CTAB method was followed to isolate plant cellular DNA from 21 days old seedlings (Murray and Thompson, 1980). Genomic DNA quality was assessed using 0.8% agarose gel electrophoresis with λ DNA as the standard and quantification was carried out with nanodrop. Parents and the population of RILs were genotyped using the 35 K SNP Axiom breeders’ array.

### Linkage map construction and QTL analysis

The linkage map generated by Manjunath et al. (2023) for the same population was used for QTL mapping. A total of 4,106 markers were reported to be polymorphic between the parents**.** 3,407 non-redundant SNP markers were utilized to create the linkage map using the IciMapping v4.2 software. The map distances between markers were calculated using the Kosambi mapping functions. The linear order of the markers was established using marker grouping using a rec value of 0.37. The function K-optimality 3-optMAP with NN initials of 10 is used for ordering. Rippling was carried out using recombination with a window size of 5 cm. QTL mapping was carried out using IciMapping 4.2 software (Meng et al., 2015) with inclusive composite interval mapping (Wang, 2009). BLUP values were calculated in the individual environment and pooled over the environment, which is used for QTLs analysis. The LOD score of 4.0 along with 1,000 permutations was chosen for the declaration of the QTLs.

### Identification of candidate genes

The candidate genes were identified based on the positions of flanking markers of the respective QTLs. BLAST search was done to identify putative candidate genes in the physical location of markers with respect to IWGSC wheat (*Triticum aestivum* L.) reference genome embedded using the Ensembl Plants database**.** The candidate genes responsible for protein coding were also confirmed using BLASTP in National Center for Biotechnology Information (NCBI) database.

## Results

### Mean squares, variability, and heritability

Two-way analysis of ANOVA indicated that there were significant genotype-treatment interactions for all traits at *p* < 0.05 (Table 1). From the one-way analysis of ANOVA, it is revealed that there was also high significant difference in staygreen traits (SPAD and LSR) and SRM (*p* < 0.001) in the mapping population under all environmental conditions viz. control, drought, heat, and combined stress respectively (Supplementary Table S1). This indicates the presence of genetic variation in the population. The range, broad sense of heritability (h^2^), genetic coefficient of variance (GCV), least significance difference (LSD), and coefficient of variation (CV) are given in Supplementary Table S2. Frequency distribution revealed the normal distribution of the traits in all conditions. Frequency distribution using histograms depicting SPAD, LSR, and SRE under all conditions are displayed in Supplementary Figure S2.

**TABLE 1:** Two-way ANOVA for staygreen and SRM traits.

Source of variations	df	LSR	SSI	SPAD_A	SPADA10	SPADA20	TGW	SRM	EWD	SRE	SSWD
ENV	1	4.440*	107.198*	223.64*	9943.79*	31,113.27*	17,325.85*	54.639*	104.9984*	35,437.29*	27,361.7*
REP (ENV)	219	0.009*	0.373*	5.05*	1.12^ns^	52.50*	10.80*	0.001*	0.0565*	3.48*	14.8*
BLOCK(REP*ENV)	20	0.002*	0.085*	5.74*	1.23^ns^	6.98*	4.49*	0.003*	0.0721*	4.57*	28.4*
GEN	199	0.003*	0.453*	32.89*	120.04*	569.99*	107.74*	0.125*	0.3929*	120.94*	141.7*
GEN: ENV		0.003*	0.335*	17.93*	111.26*	326.56*	22.54*	0.057*	0.2768*	62.68*	97.1*
Residuals		0.001	0.063	5.09	1.23	6.80	2.96	0.002	0.0494	3.04	20.6

All variables are significant (*p* < 0.05) genotype-vs-environment interaction, whereas ns represents non-significant.

The mean SPAD value at anthesis was recorded highest under combined stress conditions (48.80) followed by heat stress (47.91), while a mean SPAD value of 47.68 and 47.09 was recorded under control and drought conditions respectively. The mean SPAD value after 10 DAA was highest under control conditions followed by drought, heat, and combined stress conditions respectively. Moreover, there was a drastic reduction in SPAD values after 20 DAA under all stress conditions. Mean LSR was recorded as highest (0.46/day) under combined stress conditions, while the minimum value of LSR was recorded under control conditions (0.24/day). The mean value of SRM (1.06 mg/stem) and SRE (38.82%) was recorded as highest under drought stress conditions, while the minimum value of SRM (0.27 mg/stem) and SRE (16.99%) was recorded under heat stress conditions. The highest SSWD (34.20 mg/cm of stem) was recorded under drought conditions, while the minimum value of 15.95 mg/cm of the stem was recorded under heat-stress conditions. Similarly, EWD of 1.34 g/ear was recorded highest under drought stress, while a minimum of 0.36 g/ear was recorded under heat stress conditions. The mean TGW of 39.72 gm was recorded highest under control conditions, while the lowest TGW of 25.27 gm was recorded under combined stress conditions. The coefficient of variation was highest under combined stress conditions, while the minimum was recorded under heat stress conditions. The broad sense of heritability was recorded as medium to high under all conditions.

### Multivariate analysis

#### Correlation

**FIGURE 1:**
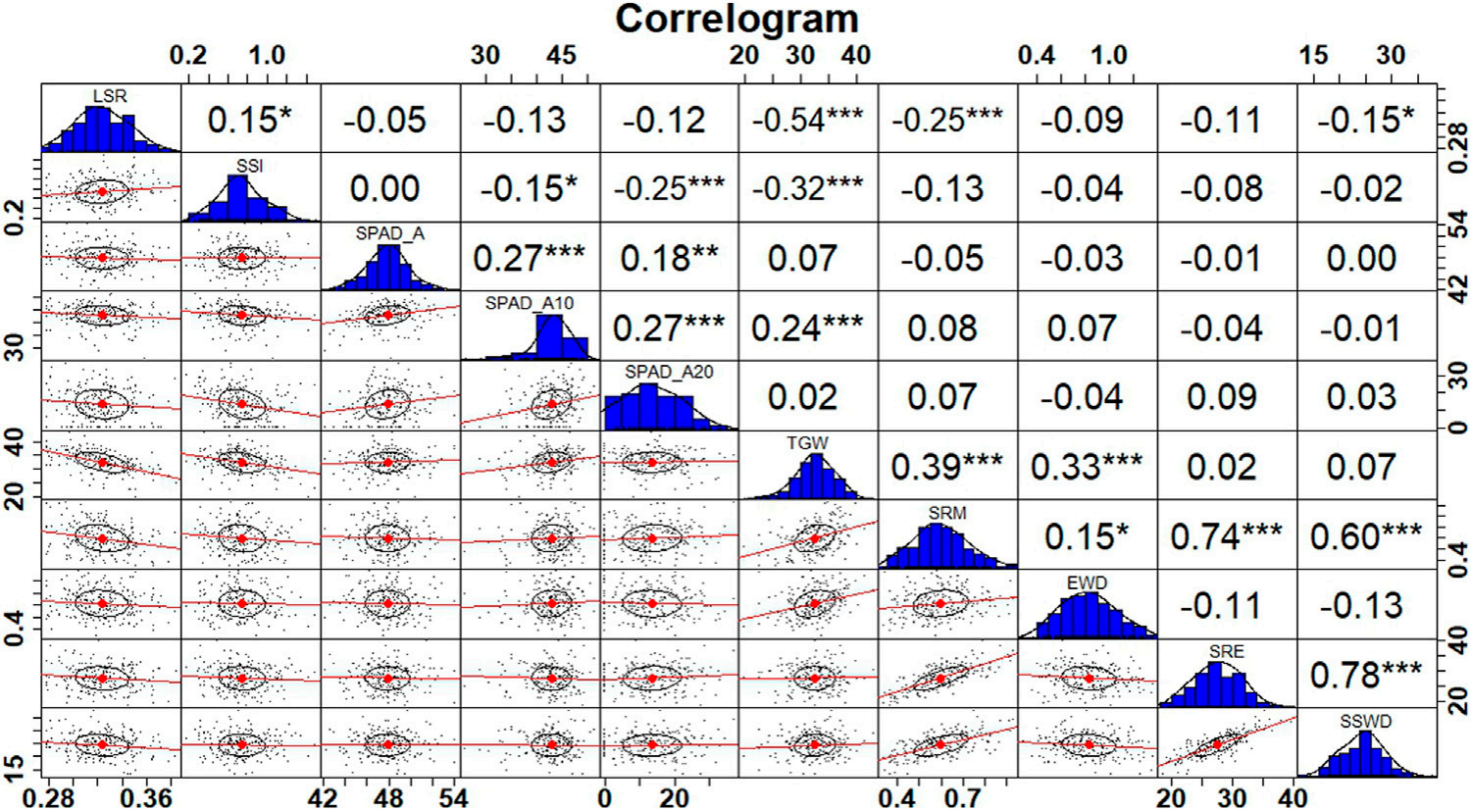
Correlogram depicting correlation coefficients between traits in pooled environments.

In pooled multi-environment using common traits in all conditions, a positive association was observed for TGW with SPAD at all stages. Moreover, a significant positive correlation of SSWD was observed with SRM (0.60^***^) and SRE (0.78^***^) at *p* < 0.001. Furthermore, a positive correlation of EWD was found with SRM (0.15^*^) at *p* < 0.05 (Figure 1).

Individually, under control conditions, a negative correlation was observed between TGW and SPAD values at all stages (Supplementary Figure S3). Furthermore, a positive correlation was observed for LSR with SPAD_A (0.22^**)^ and SPAD_A20 (0.15^*^). Moreover, a significant positive correlation of SSWD was observed with SRM (0.62^***^) and SRE (0.70^***^) at p< 0.001, and positive association was also observed for EWD with SRM (0.19^**^), SRE (0.03), and SSWD (0.01). Under drought conditions, a positive correlation was observed for TGW with SPAD_A10 and SPAD_A20 (Supplementary Figure S3). A positive correlation was observed for LSR with SPAD_A (0.12). A significantly higher association of SSWD was recorded with SRM (0.66^***^) and SRE (0.82^***^) as compared to the control condition indicating greater stem reserve mobilisation under drought stress. Under late sown heat stress conditions, a strong positive association was observed for TGW with SPAD value at all stages (Supplementary Figure S3). A notable correlation of SSWD was also observed with SRM (0.47^***^) and SRE (0.56^***^) which is the least among the stress conditions indicating that heat stress probably severely impaired the stem reserve mobilisation. A positive correlation was also observed for EWD with SRM, SRE, and SSWD respectively. Under the combined heat and drought stress condition, a positive correlation was detected for TGW with SPAD_A, SPADA-5, and SAPD_A20 respectively (Supplementary Figure S3). A higher positive association of SSWD was also noted with SRM (0.69^***^) and SRE (0.77^***^) at *p* < 0.001. A positive association of EWD with SRM and SSWD was also detected under combined stress conditions.

### Principal component analysis (PCA)

**FIGURE 2:**
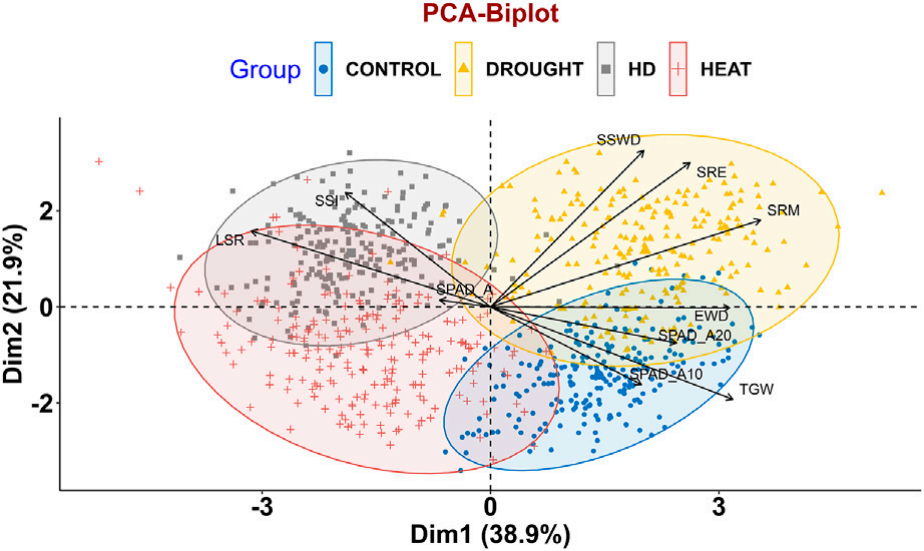
PCA biplot depicting variability of RILs population control, drought, heat and combined stress conditions.

**FIGURE 3:**
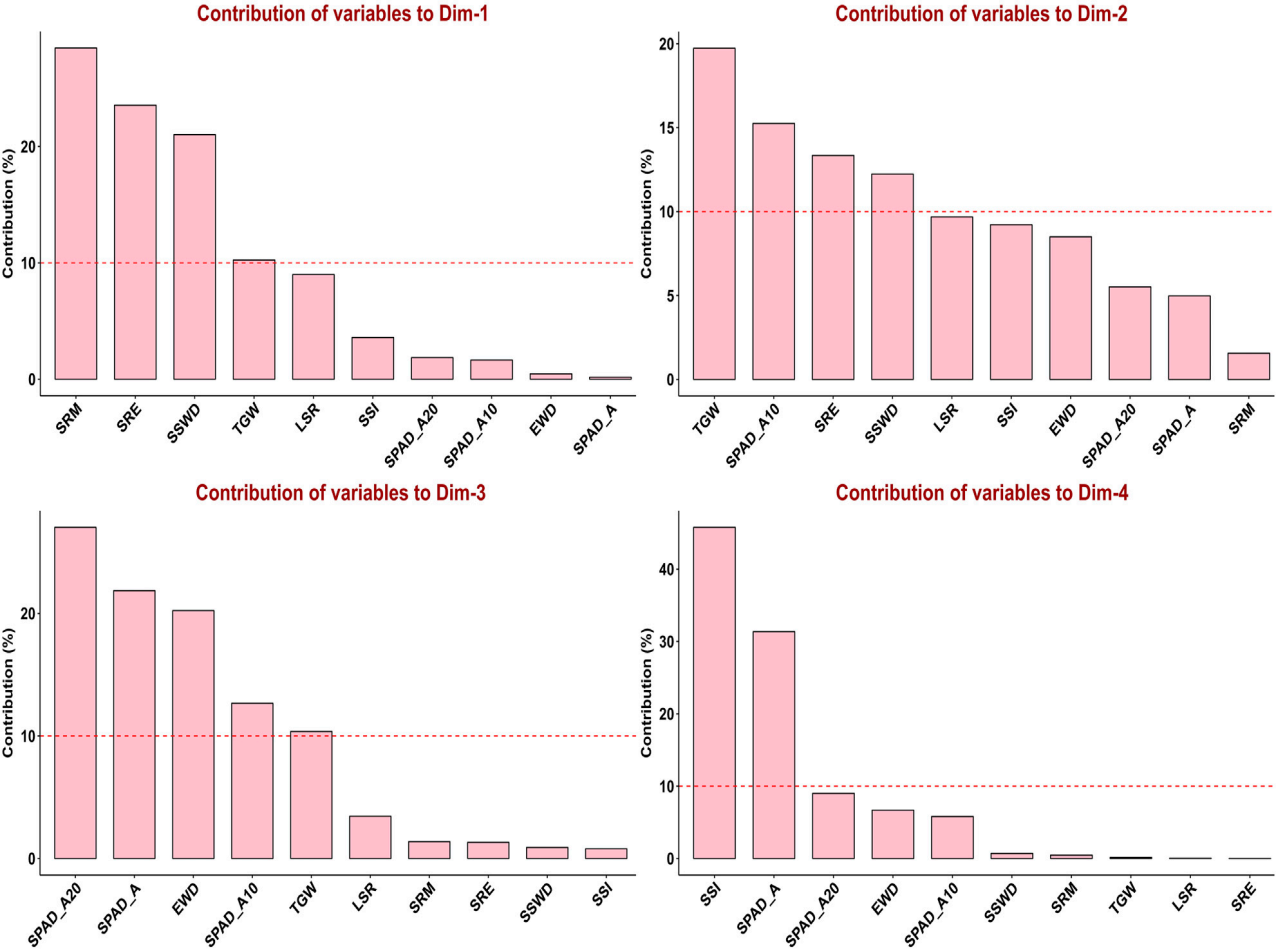
Contribution of variables to principal components in pooled environments.

In order to assess the trait variability, which is crucial for QTLs mapping, principal component analysis (PCA) was performed on multi-environmental data sets. Non-overlapping of ellipses amongst the environments suggested greater variation in the studied traits in different environmental conditions. In addition to that, no overlapping was observed between the control and combined stress condition, indicating greater variation in traits. In pooled multi-environment, dimension-1 (Dim-1) contributed 38.9% of the total variance, whereas 21.9% of the total variance was contributed by dimension-2 (Dim-2) (Figure 2). Traits like SRM, SRE, SSWD, and TGW contributed towards the Dim-1, whereas TGW, SPAD_A10, SRE and SSWD contributed towards the Dim-2. Furthermore, traits like SPAD_A20, SPAD_A, EWD, SPAD_A10 and TGW contributed towards the Dim-3. However, SSI and SPAD_A both contributed toward the Dim-4 (Figure 3).

**FIGURE 4:**
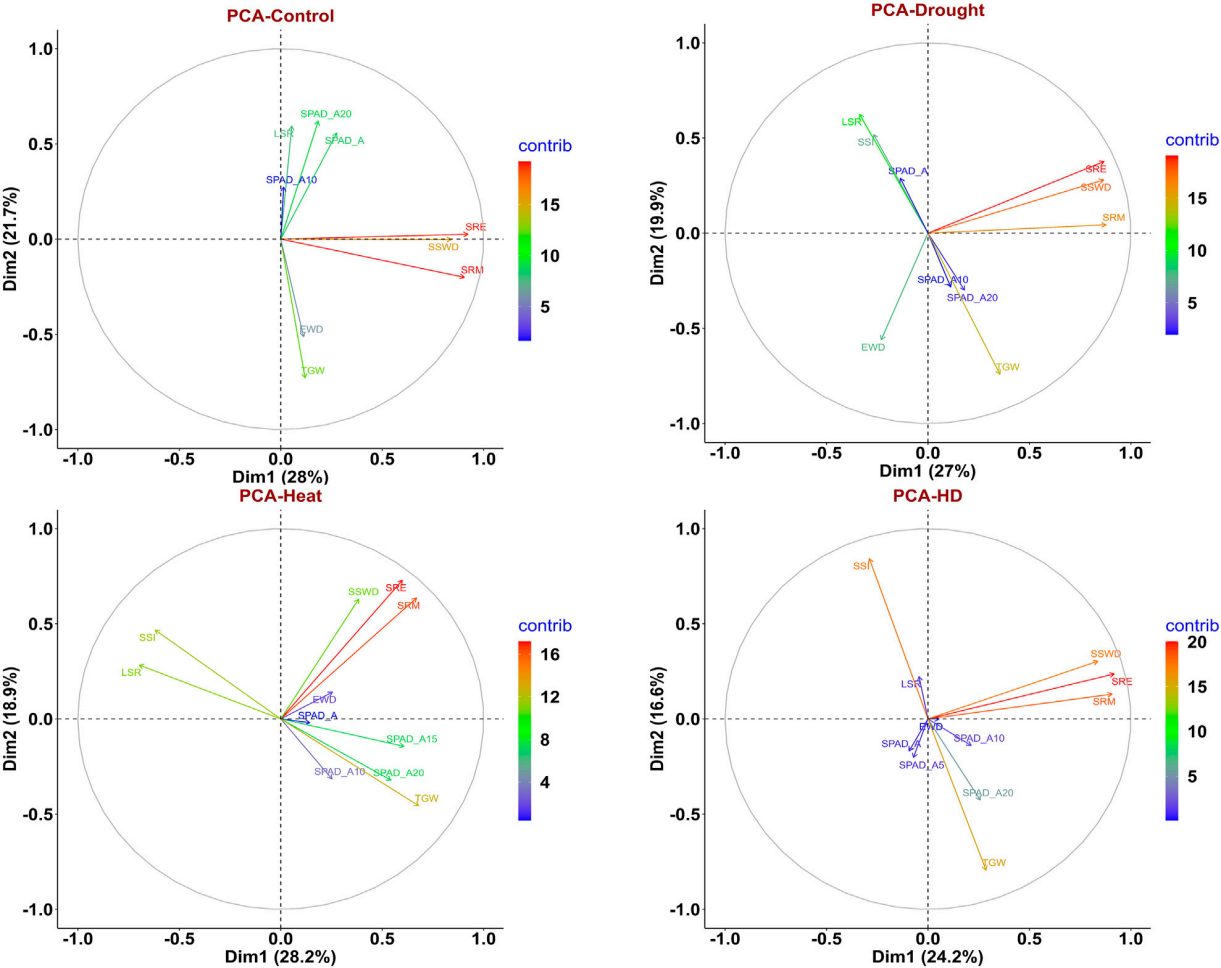
PCA biplot depicting contribution of traits to PC1 and PC2 for in control, drought, heat, and combined stress conditions.

Under the control condition, the first three principal components (eigenvalue>1) contributed 63.277% of the total variance. Dimension-1 (Dim1) contributed 28% of the total variance of data, while Dimension-2 (Dim2) contributed 21.7% of the total variance of the data. Under drought conditions (restricted irrigation), the first three principal components contributed 61.009% of the total variance. Dim1 contributed 27% of the total variance, while Dim2 19.9% of the total variance of the traits. Under the late-shown high-temperature stress, the first four components explained 69.736% of the total variance. 28.2% of the total variance was contributed by Dim1, whereas 18.9% of the total variance was contributed by Dim-2**.** Furthermore, under combined stress (heat and drought) condition, the first five components contributed about 72.321% of the total variance. Dim-1 contributed 24.2% of the total variance, whereas 16.6% of the variance was contributed by Dim-2 (Figure 4). The eigen values, percentage of variance, and cumulative variance under all conditions were given in Supplementary Table S3.

Under control condition, traits like SRE, SRM, and SSWD contributed towards the Dim1, and TGW, SPAD_A20, LSR, SPAD_A and EWD contributed towards the Dim2. However, traits like SPAD_A10, SPAD_A20, and LSR contributed to dimension-3 (Dim3) in principal component analysis (Supplementary Figure S4). Traits SPAD_A, SPAD_A10, SPAD_A20, and LSR were clustered together with acute angles indicating that positive correlation between them. Similarly, SRM, SRE, and SSWD were clustered together indicating a positive correlation between them. However, TGW and EWD were showing a positive correlation towards the Dim2. Here, LSR and SRM were not clustered together and made an angle >90° indicating that negative correlation between them (Figure 4). Under drought conditions traits like SRE, SRM, and SSWD were major contributors toward the Dim1, while traits like TGW, LSR, EWD, and SSI, were major contributors towards Dim2. SPAD_A and SPAD_A20 were major contributors to Dim3 (Supplementary Figure S4). SRM, SRE, and SSWD were clustered together with an acute angle toward Dim1, indicating a positive correlation between them, while SPAD_A, SSI, and LSR were clustered together with an acute angle, indicating a positive correlation between them. However, SPAD_A10, SPAD_A20, and TGW were positively correlated as indicated by making an acute angle between them (Figure 4). Under late sown high-temperature stress conditions SPAD_A15, SPAD_A20, SSI, TGW, SRE, and SRM were major contributors to Dim-1. Traits like SSWD, SRM, SRE, SSI, and TGW were major contributors to Dim-2. Similarly, SPAD_A, SPAD_A10, SPAD_A15, SPAD_A20 SSI, and TGW were also contributing towards the Dim-3. Traits like SPAD_A, SPAD_A15, and EWD were contributed towards the Dim-4 (Supplementary Figure S4). There was an acute angle between SRE, SRM, and SSWD indicating a positive correlation between them. However, there was a negative correlation between LSR, SSI with SPAD traits as indicated by the obtuse angle between them (Figure 4). Under combined stress, SRE, SRM, and SSWD were major contributors to Dim-1, whereas SSI, TGW, and SPAD_A20 were major contributors to Dim-2. Traits like SPAD_10, SPAD_A20, TGW, and EWD were major contributors to Dim-3, and SPAD_A, SPADA_5, EWD, and LSR were major contributor to Dim-4. Traits like LSR, SPAD_A, and EWD were major contributors toward Dim-5 (Supplementary Figure S4). Traits like SRE, SRM, SSWD, and EWD were displaying a positive correlation between them as inferred from the acute angle made between them, whereas TGW and stress susceptibility index (SSI) showed a negative correlation between them (Figure 4).

### QTL mapping and identification of candidate genes

A total of 14 QTLs were identified for studied traits under four different conditions (control, drought, heat, and combined stress) Table 2. The Logarithm of Odds (LOD) scores for studied traits varied from 4.11 to 17.88 explaining about 5.58%–15.33% traits phenotypic variation explained. Only one QTL is mapped on chromosome 6 B for SRE and two for LSR. However, eleven QTLs were mapped for SPAD value under different stress conditions. For nine QTLs, the additive effect was positive and for five QTLs, the additive effect was negative, indicating that inheritance of favorable alleles for these loci either from parents HD3086 or HI1500 respectively. The list of candidate genes associated with the QTLs region is presented in Table 3.

**TABLE 2:** QTLs identified for Staygreen traits and SRM in the RIL population under control, drought, heat, and combined stress conditions.

Traits name	Condition	QTL names	Chromosome	Position	Left marker	Right marker	LOD	PVE (%)	Add effect	Left CI	Right CI
SPAD_A	Control	QSPAD.iari.5 B.1	chr5B	636	AX-95090540	AX-94924,365	4.21	10.79	0.7369	628.5	645.5
SPADA10		QSPAD.iari.1D	chr1D	419	AX-95189357	AX-94656001	5.42	13.42	2.4252	409.5	428.5
SPAD_A20		QSPAD.iari.2D	chr2D	133	AX-94763103	AX-94432282	4.80	9.37	−4.0989	130.5	135.5
SPAD_A	Drought	QSPAD.iari.5 B.2	chr5B	632	AX-95090540	AX-94924,365	4.11	7.95	0.9698	614.5	642.5
SPAD_A	Heat	QSPAD.iari.7A.1	chr7A	324	AX-94780124	AX-95133266	4.82	9.23	1.1579	312.5	342.5
SPAD_A		QSPAD.iari.2 B	chr2B	403	AX-94465179	AX-95146413	4.22	6.98	1.0127	402.5	404.5
SPAD_A20		QSPAD.iari.6A	chr6A	452	AX-94408525	AX-94614,034	4.42	10.44	−2.9629	448.5	452.5
SPAD_A15		QSPAD.iari.3 B.1	chr3B	451	AX-94429243	AX-94763661	9.54	5.58	7.1225	447.5	452.5
SPAD_A15		QSPAD.iari.3 B.2	chr3B	455	AX-94923714	AX-94838752	17.88	11.69	−10.4139	454.5	455.5
SPAD_A	HD	QSPAD.iari.1A	chr1A	287	AX-95167291	AX-94412593	4.94	12.77	0.3447	286.5	287.5
SPAD_A		QSPAD.iari.7A.2	chr7A	150	AX-94431804	AX-94621027	4.16	11.09	−0.3091	145.5	154.5
LSR	Drought	QLSR.iari.2D	chr2D	422	AX-95134,406	AX-94713939	4.47	8.61	−0.0025	421.5	422.5
LSR	Heat	QLSR.iari.4 B	chr4B	335	AX-94957045	AX-95215762	5.16	15.33	0.0061	334.5	335.5
SRE	Control	QSRE.iari.6 B	chr6B	187	AX-94539574	AX-94589501	4.63	7.31	1.5024	179.5	192.5

**TABLE 3:** Identified marker candidate genes in QTLs region and encoded proteins.

QTLs	Chromosomes	Genes ID	Positions	Uniprot ID	Proteins
QSPAD.iari.5 B.1 QSPAD.iari.5 B.2	chr5B	TraesCS5B02G116500	5 B: 198,416,588–198,421,636	A0A1D6DFC9	leucine-rich repeat receptor-like tyrosine-protein kinase PXC3
		TraesCS5B02G047500	5 B: 53,538,071–53,540,473	A0A341V6U3	wall-associated receptor kinase 5-like
		TraesCS5B02G543300	5 B: 696,420,260–696,423,306	W5FG07	DNA repair RAD52-like protein 2, chloroplastic
QSPAD.iari.1D	chr1D	TraesCS1D02G290700	1D: 388,645,449–388,653,819	A0A1D5SYR4	calcium-dependent protein kinase 16
		TraesCS1D02G050800	1D: 30,881,098–30,882,272	A0A341PCC1	Dirigent protein 6-like
QSPAD.iari.2D	chr2D	TraesCS2D02G222600	2D: 189,600,502–189,601,505	A0A1D5V218	protein TsetseEP-like
		TraesCS2D02G217200	2D: 180,324,417–180,328,801	A0A1D5V247	mixed-linked glucan synthase 3
QSPAD.iari.7A.1	chr7A	TraesCS7A02G018200	7A: 7,727,287–7,730,698	A0A1D6BVN4	Pre-mRNA-splicing factor Cwf15/Cwc15
QSPAD.iari.2 B	chr2B	TraesCS2B02G077200	2 B: 42,276,897–42,282,176	A0A1D5UE13	prostaglandin E synthase 2-like
QSPAD.iari.6A	chr6A	TraesCS6A02G402500	6A: 610,348,775–610,352,296	A0A1D6AD57	Ubiquitin carboxyl-terminal hydrolase
		TraesCS6A02G147800	6A: 128,438,184–128,440,927	W5GCV9	Ubiquitin carboxyl-terminal hydrolase
QSPAD.iari.3 B.1	chr3B	TraesCS3B02G422800	3 B: 659,782,983–659,788,379	A0A077S642	ceramide glucosyltransferase
		TraesCS3B02G375900	3 B: 591,366,633–591,372,331	A0A1D5W9E1	Protein FATTY ACID EXPORT 3, chloroplastic
QSPAD.iari.3 B.2	chr3B	TraesCS3B02G353000	3 B: 562,727,970–562,731,330	W5D0L0	NAD(P)H dehydrogenase (quinone)
QSPAD.iari.1A	chr1A	TraesCS1A02G151400	1A: 259,994,475–259,997,900	A0A341NL01/W4ZSJ3	cyclin-dependent kinase inhibitor 4-like isoform X1
QSPAD.iari.7A.2	chr7A	TraesCS7A02G418600	7A: 610,438,735–610,441,107	A0A0X8DFF7/W5HB56	Group II HKT transporter (potassium ion transport)
		TraesCS7A02G383100	7A: 558,099,570–558,102,160	A0A341YFU7	Serine/threonine-protein kinase
		TraesCS7A02G383000	7A: 558,079,939–558,082,558	A0A341XZL3	G-type lectin S-receptor-like serine/threonine-protein kinase
QLSR.iari.2D	chr2D	TraesCS2D02G112800	2D: 62,550,720–62,552,140	A0A1D5V1J9/A0A2X0S1F0	aspartyl protease family protein 2-like isoform X1
		TraesCS2D02G106600	2D: 58,777,176–58,781,472	A0A1D5UVG7	potassium transporter 9
QLSR.iari.4 B	chr4B	TraesCS4B02G056800	4 B: 46,615,028–46,621,497	A0A1D5XRE4	Inositol-tetrakisphosphate 1-kinase
		TraesCS4B02G008300	4 B: 5,458,308–5,460,603	A0A1D5XU24	DNA polymerase epsilon subunit D-like
QSRE.iari.6 B	chr6B	TraesCS6B02G386100	6 B: 660,664,103–660,676,384	A0A1D6ABN6	serine/threonine-protein kinase 24-like isoform X1
			6 B: 660,664,103–660,676,384	A0A1D6AMY2	serine/threonine-protein kinase OSR1-like isoform X2

### Soil plant analysis development (SPAD) value

In total, 11 QTLs were mapped on different chromosomes under varied stress conditions with LOD scores varying from 4.11 to 17.88 and PVE varied from 5.58% to 13.42%. Under control conditions, three QTLs (*QSPAD.iari.5B.1*, *QSPAD. iari.1D*, *QSPAD. iari.2D*) were mapped on chromosomes 5B, 1D, and 2D respectively and one QTL (*QSPAD.iari.5B.2*) was under drought stress conditions. Here, one of the QTL (*QSPAD.iari.5B*) for SPAD was mapped on the chr5B flanked by the same marker AX-95090540 and AX-94924,365 under control and drought conditions. Additionally, a total of five QTLs were identified on chromosomes 2 B (*QSPAD.iari.2B*), 3 B (*QSPAD.iari.3B.1*, *QSPAD. iari.3B.2*), 6A (*QSPAD.iari.6A*), and 7A (*QSPAD.iari.7A.1*) under late sown heat stress condition. However, two QTLs were mapped on chromosomes 1A (*QSPAD.iari.1A*) and 7A (*QSPAD.iari.7A.2*) under combined stress conditions. Under combined stress conditions, QSPAD. iari.1A explained 12.77% phenotypic variance flanked by markers AX-95167291 and AX-94412593 with 1 cM confidence interval. Furthermore, QSPAD. iari.3 B.2 mapped in heat conditions explained 11.69% of PVE flanked by markers AX-94923714 and AX-94838752 with 1 cM CI with the highest LOD value of 17.88.

### Leaf senescence rate (LSR)

Two QTLs were mapped for LSR with LOD scores of 4.47 and 5.16. One QTL *QLSR. iari.2D* was mapped on chromosome 2D and the other QTL *QLSR. iari.4B* was on chromosome 4 B under drought and heat conditions respectively. The favorable alleles for the trait on 2D were inherited from HI1500 and those on chromosome 4 B were inherited from HD3086. The phenotypic variance explained (PVE) of 8.61% and 15.33% was recorded for the QTLs under drought and heat stress conditions respectively. *QLSR. iari.4B* explained 15.33% phenotypic variance flanked by markers AX-94957045 and AX-95215762 with the highest LOD score of 5.16 and *QLSR. iari.2D* explained 8.61% phenotypic variance flanked by markers AX-95134,406 and AX-94713939 with LOD value of 4.47. Both QTLs were present in 1 cM confidence interval. The QTL *QLSR. iari.2D* harbor two candidate genes TraesCS2D02G112800 and TraesCS2D02G106600 which code for aspartyl protease family protein and potassium transporter 9 respectively. Furthermore, other QTL *QLSR. iari.4B* is associated with two candidate genes TraesCS4B02G056800 and TraesCS4B02G008300, which code for inositol-tetrakisphosphate 1-kinase and DNA polymerase epsilon subunit D-like to regulate LSR.

### Stem reserve mobilisation efficiency (SRE)

One QTL *QSRE. iari.6B* was detected for SRE on chromosome 6 B with a LOD score of 4.63 under control conditions. One of the parents, HD3086, contributed the favorable allele for SRE. This QTL explains about 7.31% of phenotypic variance. This region is associated with one putative candidate gene TraesCS6B02G386100, which codes for serine-threonine protein kinases namely serine-threonine protein kinase 24-like, serine-threonine protein kinase OSR1-like and serine-threonine protein kinase BLUS1-like.

### Selection of superiors RILs

**FIGURE 5:**
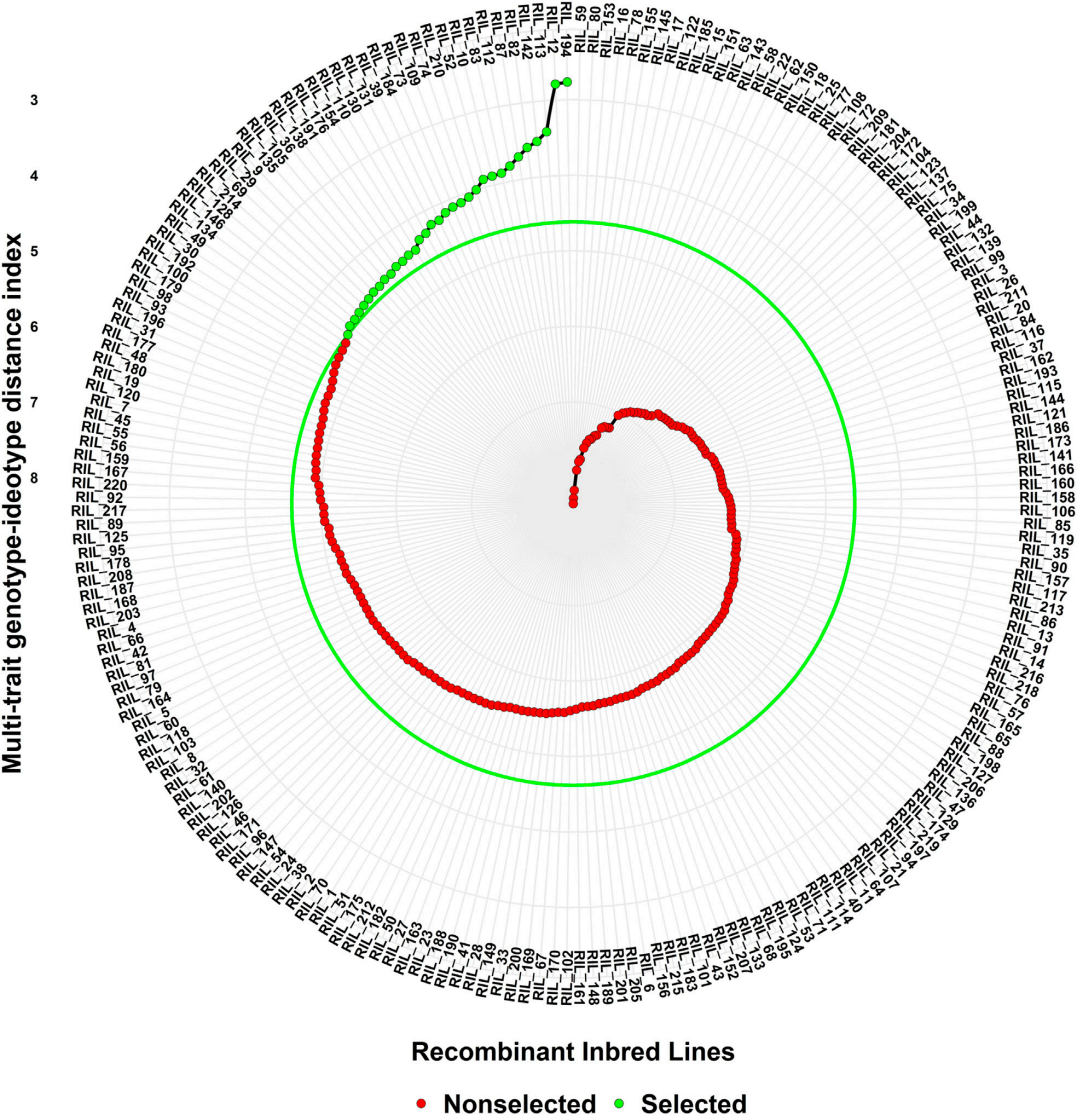
Ranking of genotypes based on MGIDI index.

Superior lines were selected based on 15% selection intensity, and lines with the lowest MGIDI values were selected as superior RILs (Figure 5). Out of 220 lines, 33 lines having both staygreen and SRM traits have been selected for further study.

## Discussion

Staygreen (SG) and stem reserve mobilisation (SRM) are two important traits that have the potential for yield enhancement under stress conditions. However, these two traits are mutually exclusive traits *i.e.,* neither trait can simultaneously contribute to grain yield. Furthermore, these traits are governed by many genes whose expression is influenced by the external environment. Therefore, the present study was to identify the QTLs regions governing both these traits (SG and SRM) as well as to identify superior lines having both traits that can contribute to grain filling under adverse climatic conditions.

The higher SPAD value at 10 DAA in control condition followed by drought, heat, and combined stress was probably because abiotic stress accelerates chlorophyll degradation (Hörtensteiner and Kräutler, 2011; Jiang et al., 2020). The reduced chlorophyll content under heat and drought stress has been reported (Raja et al., 2020). Reduction of chlorophyll content was also observed in wheat and maize under combined drought and heat stress respectively (Li et al., 2007; Anjum et al., 2017). The higher leaf senescence rate under combined stress conditions was also probably due to more chlorophyll degradation. Furthermore, this leaf senescence might be regulated by an endogenous factor, abscisic acid (ABA) which acts as a signaling molecule responding to abiotic stress during leaf senescence (Asad et al., 2019). Apart from staygreen traits, stem reserve mobilisation also contributed to grain filling under stress conditions in wheat (Blum et al., 1994).

In our present study, stem reserve mobilisation efficiency was found to be accelerated under drought conditions as compared to other stress conditions (Gurumurthy et al., 2023). It is well known that, abscisic acid (ABA) is thought to be a sensitive signal generated under water deficit stress (Davies and Zhang, 1991; Dood and Davies 2005). It has also been demonstrated that combined stem remobilisation efficiency and higher 6-fructan exohydrolase activity could contribute to grain yield enhancement under terminal drought (Joudi et al., 2012). The positive association of ABA with stem reserve mobilisation and grain filling in wheat in moderate soil drying has been also well demonstrated (Xu et al., 2016). However, the lowest SRE was recorded during the late-sown high-temperature stress condition. This might be due to the reduced expansive growth of stems (Asana and Williams, 1965; Pradhan et al., 2012) and forced maturity imposed by the high temperature stress (Djanaguiraman et al., 2018) respectively. For the selection of traits that are contributing to the yield, correlation studies are crucial. From correlation studies, when one trait is chosen to be improved, another trait can also be improved for better enhancement of crop yield.

The positive correlation of TGW with staygreen traits (SPAD value) and negative correlation with LSR indicated the significant contribution of positive traits to grain yield, which can be selected for further evaluation. A positive association of TGW with SPAD value was also reported by earlier workers (Christopher et al., 2008; Pinto et al., 2010; Lopes and Reynolds, 2012), whereas a negative association with LSR was also documented (Lu et al., 2011; Kipp et al., 2013). Similarly, a positive association of SRE with grain yield under stress conditions has also been reported (Yang et al., 2004; Sharbatkhari et al., 2016). A greater environmental effect on the expression of a specific characteristic is indicated by a lower GCV value, whereas a higher GCV value shows that an individual’s genetic makeup is primarily responsible for population variation (Manjunath et al., 2023). GCV for LSR varied from 0.2–0.32, whereas for SPAD value it varied from 2.82 to 41.23 across all stress conditions. GCV for SRE varied from 35.25–39.11 in different stress conditions. Furthermore, after choosing traits that can be targeted for yield enhancement, it is crucial to identify the chromosomal regions linked with the desired traits. Moreover, the identification of putative candidates’ genes within the identified QTLs is necessary for targeting QTLs/genes for crop improvement.

**FIGURE 6:**
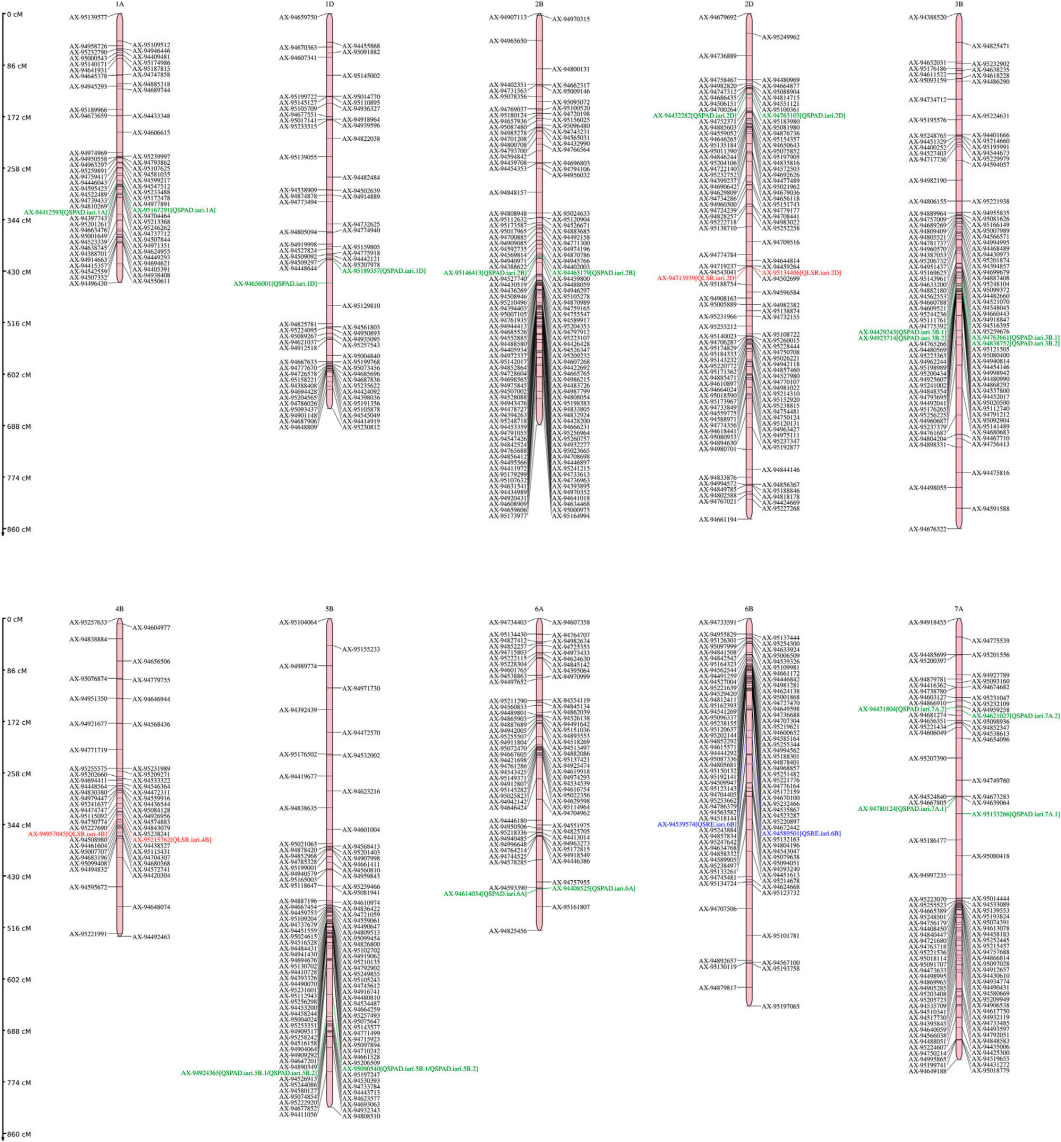
Genetic linkage map and QTL positions identified on A, B, and D genomes of RILs derived from the cross HD3086/HI1500. Green color indicates QTLs for SPAD; Red color indicates QTLs for LSR and Blue color indicates QTLs for SRE.

In the present study, 14 wheat QTLs were identified for SG and SRM, out of which 11, 2 and 1 for SPAD value, LSR, and SRE respectively (Figure 6). Out of 11 QTLs of SDAD value, 6 QTLs were mapped for major effect explained >10% phenotypic variance, whereas only one major effect QTL was mapped for LSR on chr4B. The QTL for SPAD value was also reported in earlier studies on 1A,7A, 2B, and 5 B (Peleg et al., 2009), 3 B (Kumar et al., 2012), 7A (Ilyas et al., 2014) and 1A (Tahmasebi et al., 2016) in the different mapping populations. Similarly, staygreen trait (SPAD) was also reported in chr3B in ”Chirya” X ”Sonalika” population (Kumar et al., 2010). The chromosomal regions for SPAD values encompass various genes like glucan synthase, calcium-dependent protein kinase (CDPKs), Group-II HKT transporter, and various kinases regulating signaling networks. The role of glucan in drought tolerance was also reported (Scavuzzo-Duggan et al., 2021). CDPK1 from ginger was reported to be involved in drought tolerance by retaining higher chlorophyll content in *Nicotiana tabacum* (Vivek et al., 2013). It was reported that candidate genes TraesCS3B02G353000 in *QSPAD. iari.3B.2* encodes NAD(P)H dehydrogenase which probably regulates staygreen by maintaining the balance of the redox system in the electron transfer chain in the chloroplast as well as by providing extra reducing power (ATP) for biochemical reactions (Ma et al., 2021). Mapping of *QLSR. iari.2D* affecting flag LSR detected in chr2D under drought conditions was also reported in different mapping populations (Verma et al., 2004; Barakat et al., 2013; Saleh et al., 2014). However, one major effect noble QTL *QLSR. iari.4B* was found on chr4B under late-sown high-temperature stress conditions. The putative candidate gene TraesCS2D02G106600 in the *QLSR. iari.2D* encodes the potassium transporter 9 and it has been reported that flag leaf potassium was involved in inducing drought tolerance by promoting ABA degradation, which is known to induce leaf senescence in barley (Hosseini et al., 2016). Another candidate gene TraesCS2D02G112800 in *QLSR. iari.2D* encodes aspartyl protease family protein, which is involved in the regulation of leaf senescence under stress. Aspartyl protease is one of the four large proteolytic enzyme families regulating plant growth and development (Cao et al., 2019). It is well established that protein breakdown is an important catabolic process in plants during leaf senescence with an indispensable role in nutrient recycling (Diaz-Mendoza et al., 2016). The role of aspartic protease (CND41) in the regulation of leaf senescence by degrading partially denatured RUBISCO *in vitro* was also demonstrated (Kato et al., 2004). Our studies also predicted the possible role of aspartyl protease in the regulation of leaf senescence under drought stress. The *QLSR. iari.4B* for LSR harbors one gene TraesCS4B02G056800, which codes for inositol-tetrakisphosphate 1-kinase and another gene TraesCS4B02G008300 which codes for DNA polymerase epsilon subunit D-like. The role of ABA in the regulation of phosphoinositide metabolism in plants was reported (Fleet et al., 2009; Jia et al., 2019), which in turn regulates leaf senescence. *QSRE. iari.6B* for stem reserve mobilisation efficiency was mapped on chr6B. QTLs for stem water-soluble carbohydrates (WSC) were also reported on chr6B (Yang et al., 2007). The putative candidate gene TraesCS6B02G386100 in *QSRE. iari.6B* encodes a serine-threonine protein kinase involved in many signaling networks. The role of serine-threonine protein kinase in ABA-dependent plant developmental regulation under stress has been well documented by earlier workers (Kulik et al., 2011; Yang et al., 2012; Ali et al., 2020) and ABA in turn regulated stem reserve mobilisation (Travaglia et al., 2012). Furthermore, MGIDI index has been used in many experimental conditions for the screening of genotypes by earlier workers (Benakanahalli et al., 2021; Pour-Aboughadareh and Poczai, 2021; Al-Ashkar et al., 2023).

## Conclusion

Staygreen traits and stem reserve mobilisation are the two important traits contributing to grain filling during drought and heat stress. A positive correlation of staygreen traits and SRM with their respective yield traits were found, which can be useful for the selection of superior line based on trait values in future studies. From PCA analysis, important latent variables contributing significantly to total variance can be selected for further selection by avoiding trait redundancy and multicollinearity issues. From our study, 14 wheat QTLs were identified for SG and SRE, out of which 11, 2, and 1 were for SPAD value, LSR, and SRE respectively. In-silico identification of candidate genes linked to QTLs region needs to be validated through gene expression analysis in future studies. Physio-biochemical characterization of selected lines through MGIDI can be done for further validation of identified putative candidate genes. The QTL mapping conducted in this study provides primary information on genomic regions linked to SG and SRM. Furthermore, validation of the QTLs can be carried out for further use in the MAS program.

## Data Availability

The original contributions presented in this study are included in the article/Supplementary Material. Further inquiries can be directed to the corresponding authors.

## Abbreviations

SPAD, Soil plant analysis development; LSR, Leaf senescence rate; EWD, Ear weight difference; SSWD, Stem specific weight difference; SRM, Stem reserve mobilisation; SRE, Stem reserve mobilisation efficiency; TGW, Thousand grain weight; SSI, Stress susceptibility index; MAS, Marker assisted selection.

## References

[B1] Al-Ashkar I. Sallam M. Almutairi K. F. Shady M. Ibrahim A. Alghamdi S. S. (2023). Detection of high-performance wheat genotypes and genetic stability to determine complex interplay between genotypes and environments. Agronomy 13 (2), 585. 10.3390/agronomy13020585

[B2] Ali A. Pardo J. M. Yun D. J. (2020). Desensitization of ABA-signaling: the swing from activation to degradation. Front. Plant Sci. 11, 379. 10.3389/fpls.2020.00379 32391026 PMC7188955

[B3] Anjum S. A. Tanveer M. Hussain S. Ashraf U. Khan I. Wang L. (2017). Alteration in growth, leaf gas exchange, and photosynthetic pigments of maize plants under combined cadmium and arsenic stress. Water, Air, and Soil Pollut. 228, 13–12. 10.1007/s11270-016-3187-2

[B4] Asad M. A. U. Zakari S. A. Zhao Q. Zhou L. Ye Y. Cheng F. (2019). Abiotic stresses intervene with ABA signaling to induce destructive metabolic pathways leading to death: premature leaf senescence in plants. Int. J. Mol. Sci. 20 (2), 256. 10.3390/ijms20020256 30634648 PMC6359161

[B5] Asana R. D. Williams R. F. (1965). The effect of temperature stress on grain development in wheat. Aust. J. Agric. Res. 16 (1), 1–13. 10.1071/ar9650001

[B6] Barakat M. N. Wahba L. E. Milad S. I. (2013). Molecular mapping of QTLs for wheat flag leaf senescence under water-stress. Biol. Plant. 57, 79–84. 10.1007/s10535-012-0138-7

[B7] Benakanahalli N. K. Sridhara S. Ramesh N. Olivoto T. Sreekantappa G. Tamam N. (2021). A framework for identification of stable genotypes basedon MTSI and MGDII indexes: an example in guar (*Cymopsis tetragonoloba* L). Agronomy 11 (6), 1221. 10.3390/agronomy11061221

[B8] Bennett D. Izanloo A. Reynolds M. Kuchel H. Langridge P. Schnurbusch T. (2012). Genetic dissection of grain yield and physical grain quality in bread wheat (*Triticum aestivum* L) under water-limited environments. Theor. Appl. Genet. 125, 255–271. 10.1007/s00122-012-1831-9 22374139

[B9] Bhering L. L. Laviola B. G. Salgado C. C. Sanchez C. F. B. Rosado T. B. Alves A. A. (2012). Genetic gains in physic nut using selection indexes. Pesqui. Agropecuária Bras. 47, 402–408. 10.1590/s0100-204x2012000300012

[B10] Bhunia P. Das P. Maiti R. (2020). Meteorological drought study through SPI in three drought-prone districts of West Bengal, India. Earth Syst. Environ. 4 (1), 43–55. 10.1007/s41748-019-00137-6

[B11] Blum A. Sinmena B. Mayer J. Golan G. Shpiler L. (1994). Stem reserve mobilisation supports wheat-grain filling under heat stress. Funct. Plant Biol. 21 (6), 771–781. 10.1071/pp9940771

[B12] Blum A. (1998). Improving wheat grain filling under stress by stem reserve mobilisation. Euphytica 100 (1), 77–83. 10.1023/a:1018303922482

[B13] Cao S. Guo M. Wang C. Xu W. Shi T. Tong G. (2019). Genome-wide characterization of aspartic protease (AP) gene family in *Populus trichocarpa* and identification of the potential PtAPs involved in wood formation. BMC Plant Biol. 19 (1), 1–17. 10.1186/s12870-019-1865-0 31234799 PMC6591973

[B14] Christopher J. T. Manschadi A. M. Hammer G. L. Borrell A. K. (2008). Developmental and physiological traits associated with high yield and staygreen phenotype in wheat. Aust. J. Agric. Res. 59 (4), 354–364. 10.1071/ar07193

[B15] Davies W. J. Zhang J. (1991). Root signals and the regulation of growth and development of plants in drying soil. Annu. Rev. Plant Biol. 42 (1), 55–76. 10.1146/annurev.pp.42.060191.000415

[B16] Diaz-Mendoza M. Velasco-Arroyo B. Santamaria M. E. González-Melendi P. Martinez M. Diaz I. (2016). Plant senescence and proteolysis: two processes with one destiny. Genet. Mol. Biol. 39, 329–338. 10.1590/1678-4685-GMB-2016-0015 27505308 PMC5004835

[B17] Djanaguiraman M. Boyle D. L. Welti R. Jagadish S. V. K. Prasad P. V. V. (2018). Decreased photosynthetic rate under high temperature in wheat is due to lipid desaturation, oxidation, acylation, and damage of organelles. BMC Plant Biol. 18, 55–17. 10.1186/s12870-018-1263-z 29621997 PMC5887265

[B18] Dodd I. C. Davies W. J. (2010). “Hormones and the regulation of water balance,” in Plant hormones. Editor Davies P. J. (Dordrecht: Springer). 10.1007/978-1-4020-2686-7_23

[B19] Ehdaie B. Alloush G. A. Madore M. A. Waines J. G. (2006). Genotypic variation for stem reserves and mobilization in wheat: I. Post anthesis changes in internode dry matter. Crop Sci. 46 (2), 735–746. 10.2135/cropsci2005.04-0033

[B20] El Habti A. Fleury D. Jewell N. Garnett T. Tricker P. J. (2020). Tolerance of combined drought and heat stress is associated with transpiration maintenance and water soluble carbohydrates in wheat grains. Front. Plant Sci. 11, 568693. 10.3389/fpls.2020.568693 33178236 PMC7593570

[B21] Fleet C. M. Ercetin M. E. Gillaspy G. E. (2009). Inositol phosphate signaling and gibberellic acid. Plant Signal. Behav. 4 (1), 73–74. 10.4161/psb.4.1.7418 19704714 PMC2634079

[B22] Fokar M. Blum A. Nguyen H. T. (1998). Heat tolerance in spring wheat. II. Grain filling. Euphytica 104, 9–15. 10.1023/a:1018322502271

[B23] Gurumurthy S. Arora A. Krishna H. Chinnusamy V. Hazra K. K. (2023). Genotypic capacity of post-anthesis stem reserve mobilization in wheat for yield sustainability under drought and heat stress in the subtropical region. Front. Genet. 14, 1180941. 10.3389/fgene.2023.1180941 37408776 PMC10318140

[B24] Hörtensteiner S. Kräutler B. (2011). Chlorophyll breakdown in higher plants. Biochimica Biophysica Acta (BBA)-Bioenergetics 1807 (8), 977–988. 10.1016/j.bbabio.2010.12.007 21167811

[B25] Hosseini S. A. Hajirezaei M. R. Seiler C. Sreenivasulu N. von Wirén N. (2016). A potential role of flag leaf potassium in conferring tolerance to drought-induced leaf senescence in barley. Front. Plant Sci. 7, 206. 10.3389/fpls.2016.00206 26955376 PMC4768371

[B26] Ilyas M. Ilyas N. Arshad M. Kazi A. G. Kazi A. M. Waheed A. (2014). QTL mapping of wheat doubled haploids for chlorophyll content and chlorophyll fluorescence kinetics under drought stress imposed at anthesis stage. Pak. J. Bot. 46 (5), 1889–1897.

[B27] IPCC, 2021: Climate change 2021: The physical science basis. Contribution of working Group I to the sixth assessment report of the intergovernmental panel on climate change [Masson-Delmotte V. Zhai P. Pirani A. Connors S. L. Péan C. Berger S. (eds.)]. Pp-40. Cambridge University Press.

[B28] Jahufer M. Z. Z. Casler M. D. (2015). Application of the Smith‐Hazel selection index for improving biomass yield and quality of switchgrass. Crop Sci. 55 (3), 1212–1222. 10.2135/cropsci2014.08.0575

[B29] Jia Q. Kong D. Li Q. Sun S. Song J. Zhu Y. (2019). The function of inositol phosphatases in plant tolerance to abiotic stress. Int. J. Mol. Sci. 20 (16), 3999. 10.3390/ijms20163999 31426386 PMC6719168

[B30] Jiang Z. Zhu L. Wang Q. Hou X. (2020). Autophagy-related 2 regulates chlorophyll degradation under abiotic stress conditions in Arabidopsis. Int. J. Mol. Sci. 21 (12), 4515. 10.3390/ijms21124515 32630439 PMC7350272

[B31] Joshi A. K. Kumari M. Singh V. P. Reddy C. M. Kumar S. Rane J. (2007). Stay green trait: variation, inheritance and its association with spot blotch resistance in spring wheat (*Triticum aestivum* L). Euphytica 153, 59–71. 10.1007/s10681-006-9235-z

[B32] Joudi M. Ahmadi A. Mohamadi V. Abbasi A. Vergauwen R. Mohammadi H. (2012). Comparison of fructan dynamics in two wheat cultivars with different capacities of accumulation and remobilization under drought stress. Physiol. Plant. 144 (1), 1–12. 10.1111/j.1399-3054.2011.01517.x 21895669

[B33] Kato Y. Murakami S. Yamamoto Y. Chatani H. Kondo Y. Nakano T. (2004). The DNA-binding protease, CND41, and the degradation of ribulose-1, 5-bisphosphate carboxylase/oxygenase in senescent leaves of tobacco. Planta 220, 97–104. 10.1007/s00425-004-1328-0 15252735

[B34] Kipp S. Mistele B. Schmidhalter U. (2013). Identification of stay-green and early senescence phenotypes in high-yielding winter wheat, and their relationship to grain yield and grain protein concentration using high-throughput phenotyping techniques. Funct. Plant Biol. 41 (3), 227–235. 10.1071/FP13221 32480983

[B35] Kulik A. Wawer I. Krzywińska E. Bucholc M. Dobrowolska G. (2011). SnRK2 protein kinases—Key regulators of plant response to abiotic stresses. Omics a J. Integr. Biol. 15 (12), 859–872. 10.1089/omi.2011.0091 PMC3241737 22136638

[B36] Kumar U. Joshi A. K. Kumari M. Paliwal R. Kumar S. Röder M. S. (2010). Identification of QTLs for stay green trait in wheat (*Triticum aestivum* L) in the ‘Chirya 3’×‘Sonalika’population. Euphytica 174, 437–445. 10.1007/s10681-010-0155-6

[B37] Kumar S. Sehgal S. K. Kumar U. Prasad P. V. Joshi A. K. Gill B. S. (2012). Genomic characterization of drought tolerance-related traits in spring wheat. Euphytica 186, 265–276. 10.1007/s10681-012-0675-3

[B38] Kumar R. Harikrishna H. Barman D. Ghimire O. P. Gurumurthy S. Singh P. K. (2021a). Stay-green trait serves as a yield stability attribute under combined heat and drought stress in wheat (*Triticum aestivum* L). Plant Growth Regul. 96, 67–78. 10.1007/s10725-021-00758-w

[B39] Li C. X. Feng S. L. Yun S. Jiang L. N. Lu X. Y. Hou X. L. (2007). Effects of arsenic on seed germination and physiological activities of wheat seedlings. J. Environ. Sci. 19 (6), 725–732. 10.1016/s1001-0742(07)60121-1 17969647

[B40] Lopes M. S. Reynolds M. P. (2012). Stay-green in spring wheat can be determined by spectral reflectance measurements (normalized difference vegetation index) independently from phenology. J. Exp. Bot. 63 (10), 3789–3798. 10.1093/jxb/ers071 22412185 PMC3388823

[B41] Lu Y. Hao Z. Xie C. Crossa J. Araus J. L. Gao S. (2011). Large-scale screening for maize drought resistance using multiple selection criteria evaluated under water-stressed and well-watered environments. Field Crops Res. 124, 37–45. 10.1016/j.fcr.2011.06.003

[B42] Ma M. Liu Y. Bai C. Yong J. W. H. (2021). The significance of chloroplast NAD (P) H dehydrogenase complex and its dependent cyclic electron transport in photosynthesis. Front. Plant Sci. 12, 661863. 10.3389/fpls.2021.661863 33968117 PMC8102782

[B43] Manjunath K. K. Krishna H. Devate N. B. Sunilkumar V. P. Chauhan D. Singh S. (2023). Mapping of the QTLs governing grain micronutrients and thousand kernel weight in wheat (*Triticum aestivum* L) using high density SNP markers. Front. Nutr. 10, 1105207. 10.3389/fnut.2023.1105207 36845058 PMC9950559

[B44] Meng L. Li H. Zhang L. Wang J. (2015). QTL IciMapping: integrated software for genetic linkage map construction and quantitative trait locus mapping in biparental populations. Crop J. 3 (3), 269–283. 10.1016/j.cj.2015.01.001

[B45] Mittler R. (2006). Abiotic stress, the field environment and stress combination. Trends Plant Sci. 11, 15–19. 10.1016/j.tplants.2005.11.002 16359910

[B46] Murray M. Thompson W. (1980). Rapid isolation of high molecular weight plant DNA. Nucl. Acids Res. 8 (19), 4321–4325. 10.1093/nar/8.19.4321 7433111 PMC324241

[B47] Olivoto T. Nardino M. (2021). Mgidi: toward an effective multivariate selection in biological experiments. Bioinformatics 37 (10), 1383–1389. 10.1093/bioinformatics/btaa981 33226063

[B48] Peleg Z. V. I. Fahima T. Krugman T. Abbo S. Yakir D. A. N. Korol A. B. (2009). Genomic dissection of drought resistance in durum wheat x wild emmer wheat recombinant inbreed line population. Plant, Cell. and Environ. 32 (7), 758–779. 10.1111/j.1365-3040.2009.01956.x 19220786

[B49] Pinto R. S. Reynolds M. P. Mathews K. L. McIntyre C. L. Olivares-Villegas J. J. Chapman S. C. (2010). Heat and drought adaptive QTL in a wheat population designed to minimize confounding agronomic effects. Theor. Appl. Genet. 121, 1001–1021. 10.1007/s00122-010-1351-4 20523964 PMC2938441

[B50] Pour-Aboughadareh A. Poczai P. (2021). Dataset on the use of MGIDI index in screening drought-tolerant wild wheat accessions at the early growth stage. Data Brief 36, 107096. 10.1016/j.dib.2021.107096 34307802 PMC8257987

[B51] Pradhan G. P. Prasad P. V. V. Fritz A. K. Kirkham M. B. Gill B. S. (2012). Effects of drought and high temperature stress on synthetic hexaploid wheat. Funct. Plant Biol. 39, 190–198. 10.1071/FP11245 32480773

[B52] Prasad P. V. V. Pisipati S. R. Momčilović I. Ristic Z. (2011). Independent and combined effects of high temperature and drought stress during grain filling on plant yield and chloroplast EF‐Tu expression in spring wheat. J. Agron. Crop Sci. 197 (6), 430–441. 10.1111/j.1439-037x.2011.00477.x

[B53] Raja V. Qadir S. U. Alyemeni M. N. Ahmad P. (2020). Impact of drought and heat stress individually and in combination on physio-biochemical parameters, antioxidant responses, and gene expression in *Solanum lycopersicum* . 3 Biotech. 10, 208–218. 10.1007/s13205-020-02206-4 PMC7181466 32351866

[B54] Ram K. Rajkumar S. Munjal R. (2018). Stem reserve mobilization in relation to yield under different drought and high temperature stress conditions in wheat (*Triticum aestivum* L) genotypes. Int. J. Curr. Microbiol. Appl. Sci. 7, 3695–3704. 10.20546/ijcmas.2018.704.415

[B55] Saleh M. S. Al-Doss A. A. Elshafei A. A. Moustafa K. A. Al-Qurainy F. H. Barakat M. N. (2014). Identification of new TRAP markers linked to chlorophyll content, leaf senescence, and cell membrane stability in water-stressed wheat. Biol. Plant. 58, 64–70. 10.1007/s10535-013-0351-z 25740441

[B56] Salem K. F. M. Röder M. S. Börner A. (2007). Identification and mapping quantitative trait loci for stem reserve mobilisation in wheat (*Triticum aestivum* L). Cereal Res. Commun. 35, 1367–1374. 10.1556/crc.35.2007.3.1

[B57] Scavuzzo-Duggan T. Varoquaux N. Madera M. Vogel J. P. Dahlberg J. Hutmacher R. (2021). Cell wall compositions of Sorghum bicolor leaves and roots remain relatively constant under drought conditions. Front. Plant Sci. 12, 747225. 10.3389/fpls.2021.747225 34868130 PMC8632824

[B58] Shah N. H. Paulsen G. M. (2003). Interaction of drought and high temperature on photosynthesis and grain-filling of wheat. Plant Soil 257, 219–226. 10.1023/A:1026237816578

[B59] Sharbatkhari M. Shobbar Z. S. Galeshi S. Nakhoda B. (2016). Wheat stem reserves and salinity tolerance: molecular dissection of fructan biosynthesis and remobilization to grains. Planta 244, 191–202. 10.1007/s00425-016-2497-3 27016249

[B60] Shivramakrishnan R. Vinoth R. Arora A. J. A. Y. Singh G. P. Kumar B. Singh V. P. (2016). Characterization of wheat genotypes for stay green and physiological traits by principal component analysis under drought condition. Int. J. Agric. Sci. 12 (2), 245–251. 10.15740/has/ijas/12.2/245-251

[B61] Shokat S. Großkinsky D. K. Roitsch T. Liu F. (2020). Activities of leaf and spike carbohydrate-metabolic and antioxidant enzymes are linked with yield performance in three spring wheat genotypes grown under well-watered and drought conditions. BMC Plant Biol. 20 (1), 400–419. 10.1186/s12870-020-02581-3 32867688 PMC7457523

[B62] Shokat S. Novák O. Široká J. Singh S. Gill K. S. Roitsch T. (2021). Elevated CO2 modulates the effect of heat stress responses in *Triticum aestivum* by differential expression of an isoflavone reductase-like gene. J. Exp. Bot. 72 (21), 7594–7609. 10.1093/jxb/erab247 34050754

[B63] Shokat S. Großkinsky D. K. Singh S. Liu F. (2023). Role of genetic diversity and pre-breeding traits to improve the drought and heat tolerance of bread wheat at the reproductive stage. Food Energy Secur. (In production). 10.1002/fes3.478

[B64] Simón M. R. (1999). Inheritance of flag-leaf angle, flag-leaf area and flag-leaf area duration in four wheat crosses. Theor. Appl. Genet. 98, 310–314. 10.1007/s001220051074

[B65] Singh G. P. Prabhu K. V. Singh P. K. Singh A. M. Jain N. Ramya P. (2014). HD 3086: A new wheat variety for irrigated, timely sown condition of the north western plains zone of India. J. Wheat Res. 6 (2), 179–180.

[B66] Tahmasebi S. Heidari B. Pakniyat H. McIntyre C. L. (2016). Mapping QTLs associated with agronomic and physiological traits under terminal drought and heat stress conditions in wheat (*Triticum aestivum* L). Genome 60 (1), 26–45. 10.1139/gen-2016-0017 27996306

[B67] Talaat N. B. (2021). Polyamine and nitrogen metabolism regulation by melatonin and salicylic acid combined treatment as a repressor for salt toxicity in wheat (*Triticum aestivum* L) plants. Plant Growth Regul. 95 (3), 315–329. 10.1007/s10725-021-00740-6

[B68] Travaglia C. Balboa G. Espósito G. Reinoso H. (2012). ABA action on the production and redistribution of field-grown maize carbohydrates in semiarid regions. Plant Growth Regul. 67, 27–34. 10.1007/s10725-012-9657-7

[B69] Tricker P. J. ElHabti A. Schmidt J. Fleury D. (2018). The physiological and genetic basis of combined drought and heat tolerance in wheat. J. Exp. Bot. 69 (13), 3195–3210. 10.1093/jxb/ery081 29562265

[B70] Ulfat A. Shokat S. Li X. Fang L. Großkinsky D. K. Majid S. A. (2021). Elevated carbon dioxide alleviates the negative impact of drought on wheat by modulating plant metabolism and physiology. Agric. Water Manag. 250, 106804. 10.1016/j.agwat.2021.106804

[B71] Valipour M. Bateni S. M. Jun C. (2021). Global surface temperature: A new insight. Climate 9 (5), 81. 10.3390/cli9050081

[B72] Verma V. Foulkes M. J. Worland A. J. Sylvester-Bradley R. Caligari P. D. S. Snape J. W. (2004). Mapping quantitative trait loci for flag leaf senescence as a yield determinant in winter wheat under optimal and drought-stressed environments. Euphytica 135, 255–263. 10.1023/b:euph.0000013255.31618.14

[B73] Vijayalakshmi K. Fritz A. K. Paulsen G. M. Bai G. Pandravada S. Gill B. S. (2010). Modeling and mapping QTL for senescence-related traits in winter wheat under high temperature. Mol. Breed. 26 (2), 163–175. 10.1007/s11032-009-9366-8

[B74] Vivek P. J. Tuteja N. Soniya E. V. (2013). CDPK1 from ginger promotes salinity and drought stress tolerance without yield penalty by improving growth and photosynthesis in *Nicotiana tabacum* . PLoS One 8 (10), e76392. 10.1371/journal.pone.0076392 24194837 PMC3806807

[B75] Wang J. (2009). Inclusive composite interval mapping of quantitative trait genes. Acta Agron. Sin. 35 (2), 239–245. 10.3724/sp.j.1006.2009.00239

[B76] Xu Y. Zhang W. Ju C. Li Y. Yang J. Zhang J. (2016). Involvement of abscisic acid in fructan hydrolysis and starch biosynthesis in wheat under soil drying. Plant Growth Regul. 80, 265–279. 10.1007/s10725-016-0164-0

[B77] Yang J. Zhang J. (2006). Grain filling of cereals under soil drying. New Phytol. 169 (2), 223–236. 10.1111/j.1469-8137.2005.01597.x 16411926

[B78] Yang J. Zhang J. Wang Z. Zhu Q. Liu L. (2001a). Water deficit–induced senescence and its relationship to the remobilization of pre‐stored carbon in wheat during grain filling. Agron. J. 93 (1), 196–206. 10.2134/agronj2001.931196x

[B79] Yang J. Zhang J. Wang Z. Zhu Q. Wang W. (2001b). Hormonal changes in the grains of rice subjected to water stress during grain filling. Plant physiol. 127 (1), 315–323. 10.1104/pp.127.1.315 11553759 PMC117987

[B80] Yang J. Zhang J. Wang Z. Zhu Q. Liu L. (2004). Activities of fructan-and sucrose-metabolizing enzymes in wheat stems subjected to water stress during grain filling. Planta 220, 331–343. 10.1007/s00425-004-1338-y 15290295

[B81] Yang D. L. Jing R. L. Chang X. P. Li W. (2007). Identification of quantitative trait loci and environmental interactions for accumulation and remobilization of water-soluble carbohydrates in wheat (*Triticum aestivum* L) stems. Genetics 176 (1), 571–584. 10.1534/genetics.106.068361 17287530 PMC1893045

[B82] Yang L. Ji W. Gao P. Li Y. Cai H. Bai X. (2012). GsAPK, an ABA-activated and calcium-independent SnRK2-type kinase from G. soja, mediates the regulation of plant tolerance to salinity and ABA stress. PLoS One 7 (3), e33838. 10.1371/journal.pone.0033838 22439004 PMC3306294

[B83] Yang D. Li M. Liu Y. Chang L. Cheng H. Chen J. (2016). Identification of quantitative trait loci and water environmental interactions for developmental behaviors of leaf greenness in wheat. Front. Plant Sci. 7, 273. 10.3389/fpls.2016.00273 27014298 PMC4782216

[B84] Yang H. Hu W. Zhao J. Huang X. Zheng T. Fan G. (2021). Genetic improvement combined with seed ethephon priming improved grain yield and drought resistance of wheat exposed to soil water deficit at tillering stage. Plant Growth Regul. 95 (3), 399–419. 10.1007/s10725-021-00749-x

[B85] Zhang J. Fengler K. A. Van Hemert J. L. Gupta R. Mongar N. Sun J. (2019). Identification and characterization of a novel stay‐green QTL that increases yield in maize. Plant Biotechnol. J. 17 (12), 2272–2285. 10.1111/pbi.13139 31033139 PMC6835130

