# Supplementary material for: Multivariate analysis and genetic dissection of staygreen and stem reserve mobilisation under combined drought and heat stress in wheat (Triticum aestivum L.)
Source: Front Genet. 2023 Aug 29;14:1242048. doi: 10.3389/fgene.2023.1242048 (PMC10496116; doi:10.3389/fgene.2023.1242048)
Supplement: Supplementary file 1 [file Table1.docx]

| Control | | | | | | | | | | |
| --- | --- | --- | --- | --- | --- | --- | --- | --- | --- | --- |
| Source of Variations | Degree of freedom | LSR | SPAD_A | SPADA10 | SPADA20 | TGW | SRM | EWD | SRE | SSWD |
| Replication | 1 | 0.00001485 | 9.6318 | 0.007 | 3.03 | 0.048 | 0.000075 | 0.19525 | 3.836 | 0.131 |
| Treatments/RILs | 219 | 0.00036645*** | 17.0894*** | 54.82*** | 557.16*** | 36.484*** | 0.082408*** | 0.49987*** | 75.765*** | 111.079*** |
| Block/Rep | 20 | 0.00007822 | 8.8216 | 0.047 | 4.65 | 1.545 | 0.003458 | 0.11722 | 2.451 | 31.811 |
| Error | 199 | 0.00009801 | 6.2277 | 0.05 | 4.59 | 2.464 | 0.003511 | 0.08282 | 3.488 | 28.519 |

*** = significant at 0.1% level

Supplementary Table 1A- Mean squares of staygreen traits and SRE under control condition.

| Drought | | | | | | | | | | | |
| --- | --- | --- | --- | --- | --- | --- | --- | --- | --- | --- | --- |
| Source of Variations | Degree of freedom | LSR | SSI | SPAD_A | SPADA10 | SPADA20 | TGW | SRM | EWD | SRE | SSWD |
| Replication | 1 | 0.00091098** | 1.42497** | 3.7833 | 0.626 | 3.03 | 30.899** | 0.001646 | 0.02974 | 0.123 | 5.438 |
| Treatments/RILs | 219 | 0.00038295*** | 0.63716*** | 27.3002*** | 83.831*** | 557.16*** | 34.644*** | 0.16066*** | 0.40998*** | 83.606*** | 113.45*** |
| Block/Rep | 20 | 0.0001775* | 0.27361 | 7.2043 | 0.926 | 4.65 | 4.892* | 0.009147 | 0.15743 | 10.274** | 48.04 |
| Error | 199 | 0.00009758 | 0.20054 | 4.6904 | 0.9 | 4.59 | 2.848 | 0.005868 | 0.0999 | 5.104 | 29.656 |

*** = significant at 0.1% level, ** = significant at 1% level, * = significant at 5 % level

Supplementary Table 1B- Mean squares of staygreen traits and SRE under drought stress condition.

| Heat | | | | | | | | | | | | |
| --- | --- | --- | --- | --- | --- | --- | --- | --- | --- | --- | --- | --- |
| Source of Variations | Degree of freedom | LSR | SSI | SPADA | SPAD(A+10) | SPADA15 | SPAD(A+20) | TGW | SRM | EWD | SRE | SSWD |
| Replication | 1 | 0.0072002*** | 0.00188 | 0.2227 | 0 | 1.98 | 1.478 | 0.265 | 0.0000012 | 0.00027 | 0.007 | 2.062 |
| Treatments/RILs | 219 | 0.00175*** | 0.69781*** | 27.3149*** | 274.259*** | 441.21*** | 278.557*** | 75.432*** | 0.0292918*** | 0.151338*** | 75.17*** | 115.32*** |
| Block/Rep | 20 | 0.0005394 | 0.01487 | 0.1894 | 0.085 | 13.74 | 1.509 | 0.789 | 0.0000076 | 0.000175 | 0.023 | 0.927 |
| Error | 199 | 0.0003576 | 0.02325 | 0.193 | 0.073 | 23.02 | 1.514 | 1.132 | 0.0000072 | 0.000186 | 0.021 | 1.04 |

*** = significant at 0.1% level, ** = significant at 1% level, * = significant at 5 % level

Supplementary Table 1C- Mean squares of staygreen traits and SRE under heat stress condition.

| Combined stress (HD) | | | | | | | | | | | | |
| --- | --- | --- | --- | --- | --- | --- | --- | --- | --- | --- | --- | --- |
| Source of Variations | Degree of freedom | LSR | SSI | SPAD_A | SPAD_A5 | SPAD(A+10) | SPAD(A+20) | TGW | SRM | EWD | SRE | SSWD |
| Replication | 1 | 0.028979** | 0.067359 | 6.5685 | 5.3681 | 3.858 | 205.501** | 11.979 | 0.0040219 | 0.000716 | 9.967 | 51.645 |
| Treatments/RILs | 219 | 0.0104425*** | 0.124191*** | 14.9764*** | 17.3637*** | 40.905*** | 253.933*** | 28.7941*** | 0.0268376*** | 0.162158*** | 74.431*** | 93.326*** |
| Block/Rep | 20 | 0.0075191* | 0.051395* | 6.7264 | 13.7202 | 3.85 | 21.752 | 10.7308** | 0.0020224* | 0.013688 | 5.52 | 32.886 |
| Error | 199 | 0.0042174 | 0.029954 | 9.2601 | 18.9494 | 3.906 | 21.071 | 5.3912 | 0.0011738 | 0.01461 | 3.531 | 23.328 |

*** = significant at 0.1% level, ** = significant at 1% level, * = significant at 5 % level

Supplementary Table 1D-Mean squares of staygreen traits and SRE under combined stress (HD) condition.

| Conditions | Traits | Grand Mean | Range | LSD | CV | GCV | h^2^ |
| --- | --- | --- | --- | --- | --- | --- | --- |
| Control | LSR | 0.24 | 0.2-0.273 | 0.02 | 4.07 | 0.2 | 0.74 |
|  | SPAD_A | 47.68 | 39.30-55.80 | 3.93 | 5.22 | 5.22 | 0.63 |
|  | SPAD_A10 | 47.33 | 33.40-58.30 | 0.44 | 0.47 | 27.39 | 1.00 |
|  | SPAD_A20 | 20.81 | 0-54.50 | 0.17 | 0.41 | 230.00 | 1.00 |
|  | TGW | 39.72 | 25.6- 50.20 | 2.94 | 3.88 | 17.05 | 0.93 |
|  | SRM | 0.67 | 0.20 - 1.23 | 0.11 | 8.88 | 0.04 | 0.96 |
|  | EWD | 1.15 | 0.013 - 2.62 | 0.52 | 25.01 | 0.21 | 0.83 |
|  | SRE | 25.97 | 8.32 - 41.47 | 3.55 | 7.09 | 36.19 | 0.96 |
|  | SSWD | 20.63 | 0.37 - 44.68 | 9.11 | 25.81 | 40.97 | 0.74 |

Supplementary Table 2A- Descriptive statistics and heritability under control condition

| Conditions |  | Traits | Grand Mean | Range | LSD | CV | GCV | h^2^ |
| --- | --- | --- | --- | --- | --- | --- | --- | --- |
| Drought |  | LSR | 0.26 | 0.205-0.286 | 0.02 | 3.82 | 0.25 | 0.74 |
|  |  | SSI | 0.98 | 0.021-3.021 | 0.74 | 45.82 | 0.21 | 0.68 |
|  |  | SPAD_A | 47.09 | 36.20- 57.80 | 3.95 | 4.67 | 11.26 | 0.82 |
|  |  | SPAD_A10 | 45.17 | 27.70- 60.70 | 1.86 | 2.10 | 41.23 | 0.99 |
|  |  | SPAD_A20 | 20.45 | 0 - 53.70 | 4.21 | 10.47 | 276.27 | 0.99 |
|  |  | TGW | 35.29 | 22.30- 46.80 | 3.24 | 4.76 | 15.64 | 0.92 |
|  |  | SRM | 1.06 | 0.377 - 1.845 | 0.15 | 7.20 | 0.08 | 0.96 |
|  |  | EWD | 1.34 | 0.223 - 2.737 | 0.55 | 23.65 | 0.15 | 0.75 |
|  |  | SRE | 38.82 | 25.120-56.927 | 4.42 | 5.83 | 39.11 | 0.94 |
|  |  | SSWD | 34.20 | 9.276 - 55.301 | 9.34 | 15.97 | 41.47 | 0.74 |

Supplementary Table 2B- Descriptive statistics and heritability under drought stress condition

| Conditions | Traits | Grand Mean | Range | LSD | CV | GCV | h^2^ |
| --- | --- | --- | --- | --- | --- | --- | --- |
| Heat | LSR | 0.33 | 0.259 -0.435 | 0.03 | 5.64 | 0.32 | 0.79 |
|  | SSI | 0.99 | 0.060 - 4.114 | 0.29 | 15.08 | 0.34 | 0.97 |
|  | SPAD_A | 47.91 | 37.60 -56.70 | 0.86 | 0.92 | 13.56 | 0.99 |
|  | SPAD(A+10) | 43.06 | 0 - 58.00 | 0.53 | 0.62 | 137.05 | 1.00 |
|  | SPAD(A+20) | 7.97 | 0 -42.80 | 2.42 | 15.35 | 138.36 | 0.99 |
|  | TGW | 30.07 | 0 -44.80 | 2.05 | 3.49 | 37.17 | 0.99 |
|  | SRM | 0.27 | 0.045 - 0.582 | 0.01 | 1.01 | 0.01 | 1.00 |
|  | EWD | 0.36 | 0.008 - 1.120 | 0.03 | 3.72 | 0.08 | 1.00 |
|  | SRE | 16.99 | 4.174 -32.835 | 0.29 | 0.86 | 37.57 | 1.00 |
|  | SSWD | 15.95 | 0.240 - 38.300 | 1.99 | 6.36 | 57.14 | 0.99 |

Supplementary Table 2C-Descriptive statistics and heritability under heat stress condition

| Conditions | Traits | Grand Mean | Range | LSD | CV | GCV | h^2^ |
| --- | --- | --- | --- | --- | --- | --- | --- |
| HD | LSR | 0.46 | 0.233 - 0.625 | 0.10 | 14.09 | 0.31 | 0.59 |
|  | SSI | 0.99 | 0.097 - 1.722 | 0.30 | 17.66 | 0.05 | 0.75 |
|  | SPAD_A | 48.80 | 39.40 - 58.40 | 3.68 | 6.12 | 2.82 | 0.39 |
|  | SPAD_A10 | 36.35 | 20.60 - 50.60 | 3.71 | 5.39 | 18.26 | 0.90 |
|  | SPAD_A20 | 4.57 | 0 - 49.90 | 8.68 | 100.63 | 116.40 | 0.92 |
|  | TGW | 25.27 | 15.40 - 38.70 | 4.22 | 9.27 | 11.51 | 0.81 |
|  | SRM | 0.39 | 0.138- 0.709 | 0.07 | 8.69 | 0.01 | 0.96 |
|  | EWD | 0.46 | 0.002 - 1.270 | 0.23 | 26.37 | 0.07 | 0.91 |
|  | SRE | 28.41 | 11.089 - 44.251 | 3.68 | 6.60 | 35.25 | 0.95 |
|  | SSWD | 26.63 | 6.215 - 44.246 | 8.34 | 18.29 | 34.72 | 0.75 |

Supplementary Table 2D-Descriptive statistics and heritability under combined stress (HD) condition

| Condition | Components | Eigen value | Percentage of variance | Cumulative variance |
| --- | --- | --- | --- | --- |
| Control | 1 | 2.517 | 27.967 | 27.967 |
|  | 2 | 1.954 | 21.714 | 49.681 |
|  | 3 | 1.224 | 13.596 | 63.277 |
|  | 4 | 0.903 | 10.029 | 73.306 |
|  | 5 | 0.750 | 8.338 | 81.644 |
|  | 6 | 0.641 | 7.120 | 88.764 |
|  | 7 | 0.512 | 5.689 | 94.453 |
|  | 8 | 0.376 | 4.181 | 98.634 |
|  | 9 | 0.123 | 1.366 | 100.000 |
| Condition | Components | Eigen value | Percentage of variance | Cumulative variance |
| Drought | 1 | 2.699 | 26.989 | 26.989 |
|  | 2 | 1.994 | 19.937 | 46.927 |
|  | 3 | 1.408 | 14.082 | 61.009 |
|  | 4 | 0.971 | 9.710 | 70.719 |
|  | 5 | 0.826 | 8.256 | 78.974 |
|  | 6 | 0.779 | 7.793 | 86.767 |
|  | 7 | 0.563 | 5.628 | 92.395 |
|  | 8 | 0.370 | 3.703 | 96.098 |
|  | 9 | 0.258 | 2.580 | 98.678 |
|  | 10 | 0.132 | 1.322 | 100.000 |
| Condition | Components | Eigen value | Percentage of variance | Cumulative variance |
| Heat | 1 | 3.222 | 29.288 | 29.288 |
|  | 2 | 2.029 | 18.449 | 47.737 |
|  | 3 | 1.407 | 12.791 | 60.529 |
|  | 4 | 1.013 | 9.207 | 69.736 |
|  | 5 | 0.894 | 8.124 | 77.860 |
|  | 6 | 0.640 | 5.817 | 83.677 |
|  | 7 | 0.583 | 5.301 | 88.978 |
|  | 8 | 0.488 | 4.436 | 93.414 |
|  | 9 | 0.442 | 4.018 | 97.432 |
|  | 10 | 0.201 | 1.827 | 99.258 |
|  | 11 | 0.082 | 0.742 | 100.000 |
| Condition | Components | Eigen value | Percentage of variance | Cumulative variance |
| HD | 1 | 2.663 | 24.205 | 24.205 |
|  | 2 | 1.824 | 16.583 | 40.788 |
|  | 3 | 1.364 | 12.398 | 53.187 |
|  | 4 | 1.092 | 9.926 | 63.112 |
|  | 5 | 1.013 | 9.208 | 72.321 |
|  | 6 | 0.947 | 8.605 | 80.926 |
|  | 7 | 0.910 | 8.274 | 89.200 |
|  | 8 | 0.588 | 5.347 | 94.547 |
|  | 9 | 0.294 | 2.675 | 97.223 |
|  | 10 | 0.215 | 1.957 | 99.180 |
|  | 11 | 0.090 | 0.820 | 100.000 |

Supplementary Table 3- Eigen value, percentage of variance and cumulative variance under control, drought stress, heat stress & combined stress conditions
