# Supplementary material for: Multivariate analysis and genetic dissection of staygreen and stem reserve mobilisation under combined drought and heat stress in wheat (Triticum aestivum L.)
Source: Front Genet. 2023 Aug 29;14:1242048. doi: 10.3389/fgene.2023.1242048 (PMC10496116; doi:10.3389/fgene.2023.1242048)
Supplement: Supplementary file 2 [file Image1.pdf]

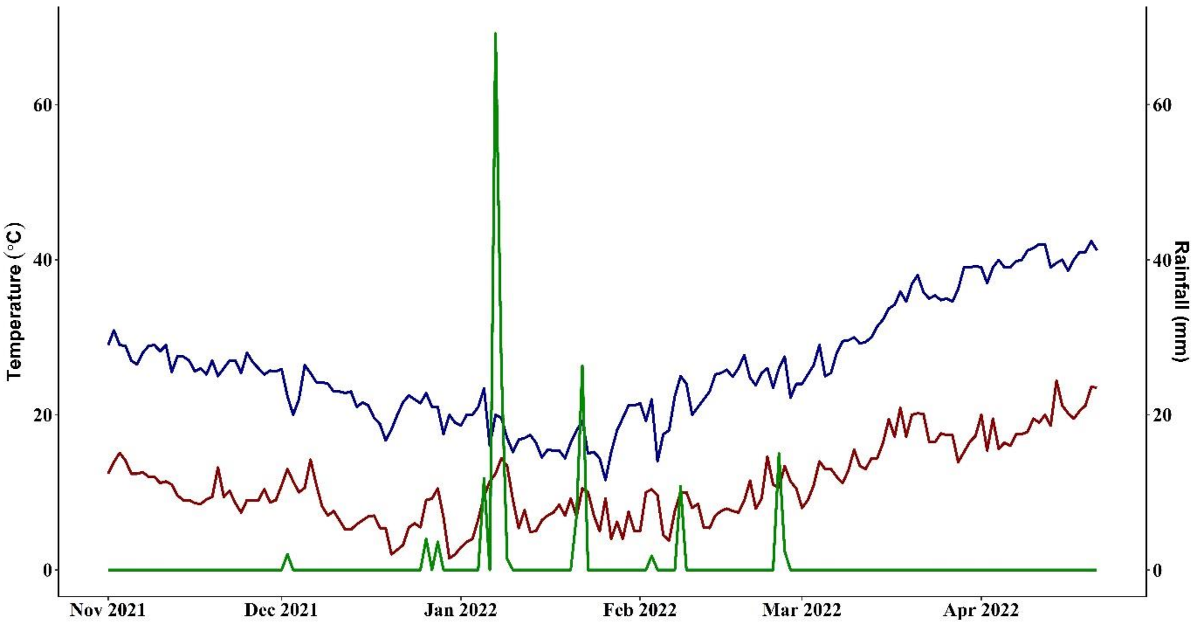


Fig 1- Weather parameters depicting maximum temperature (blue lines), minimum temperature (red line), and rainfall (green line) during 2021-22.

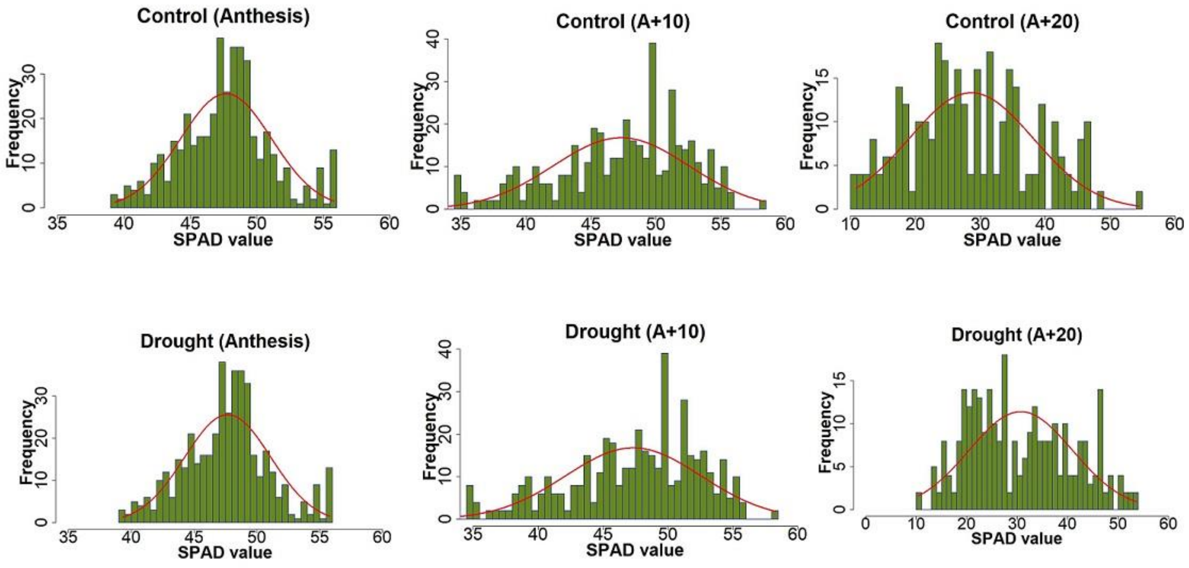


Figure 2A- Frequency distribution depicting SPAD values at anthesis, 10 DAA & 20 DAA under control & drought stress conditions.

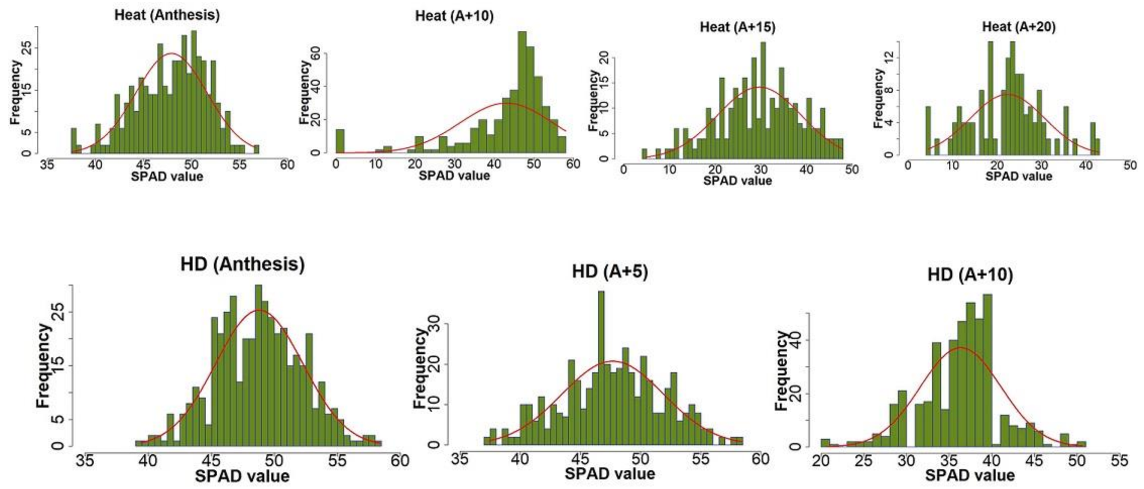


Figure 2B- - Frequency distribution depicting SPAD values at anthesis, 10 DAA, 15 DAA, 20 DAA under heat stress, & at anthesis, 5 DAA and 10 DAA under combined stress (HD) conditions.

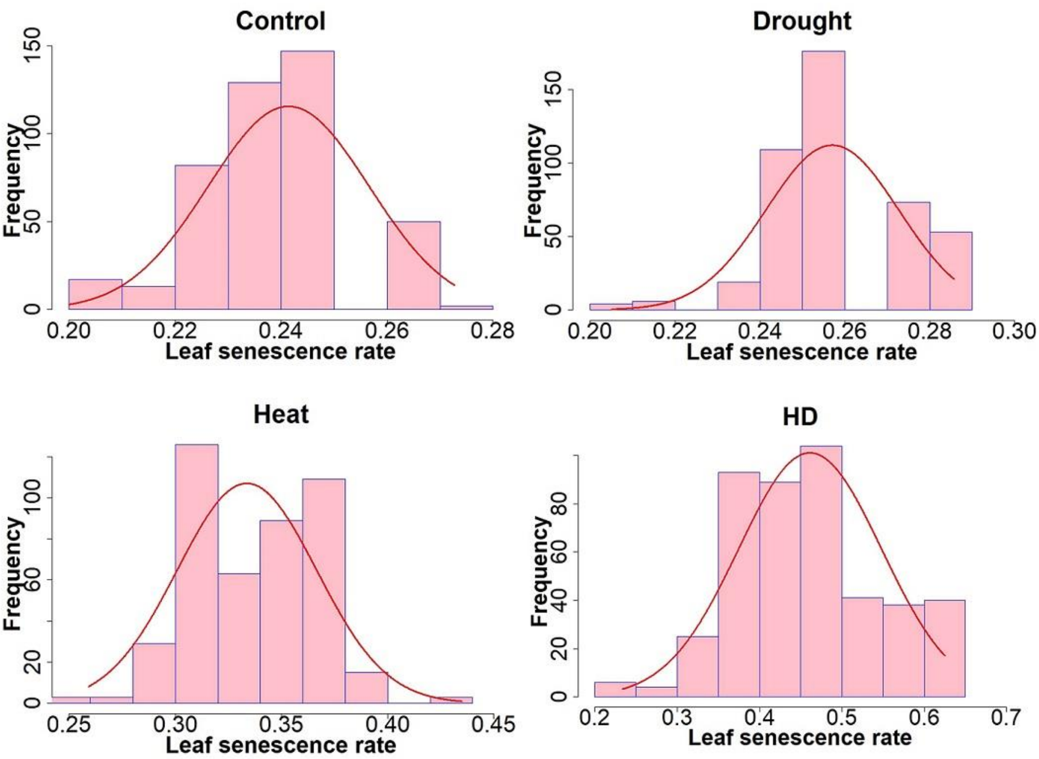


Figure 2C- Frequency distribution depicting leaf senescence rate (LSR) under control, drought stress, heat stress, and combined stress (HD) conditions.

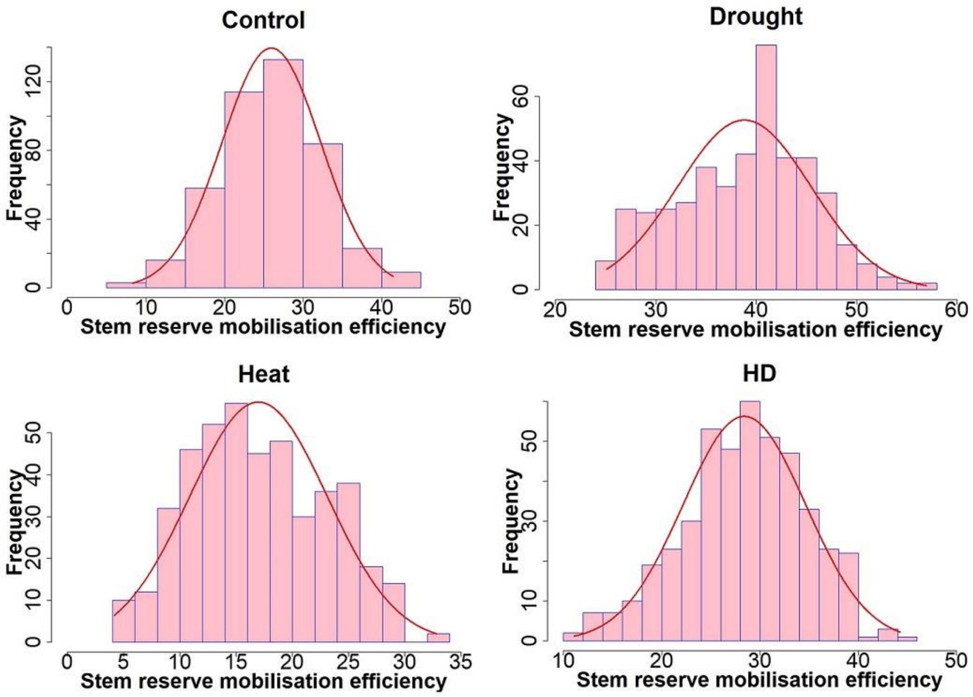


Fig 2D- Frequency distribution depicting stem reserve mobilisation efficiency (SRE) under control, drought stress, heat stress, and combined stress (HD) conditions.

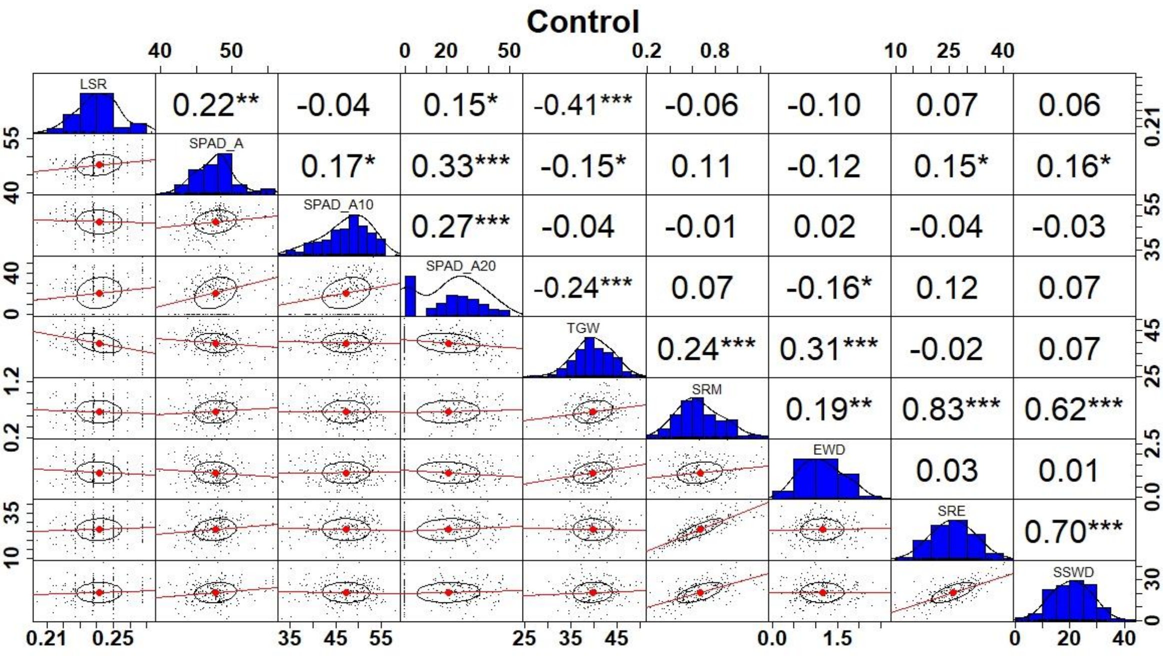


Figure 3A- Correlogram depicting correlation coefficients between traits under control condition

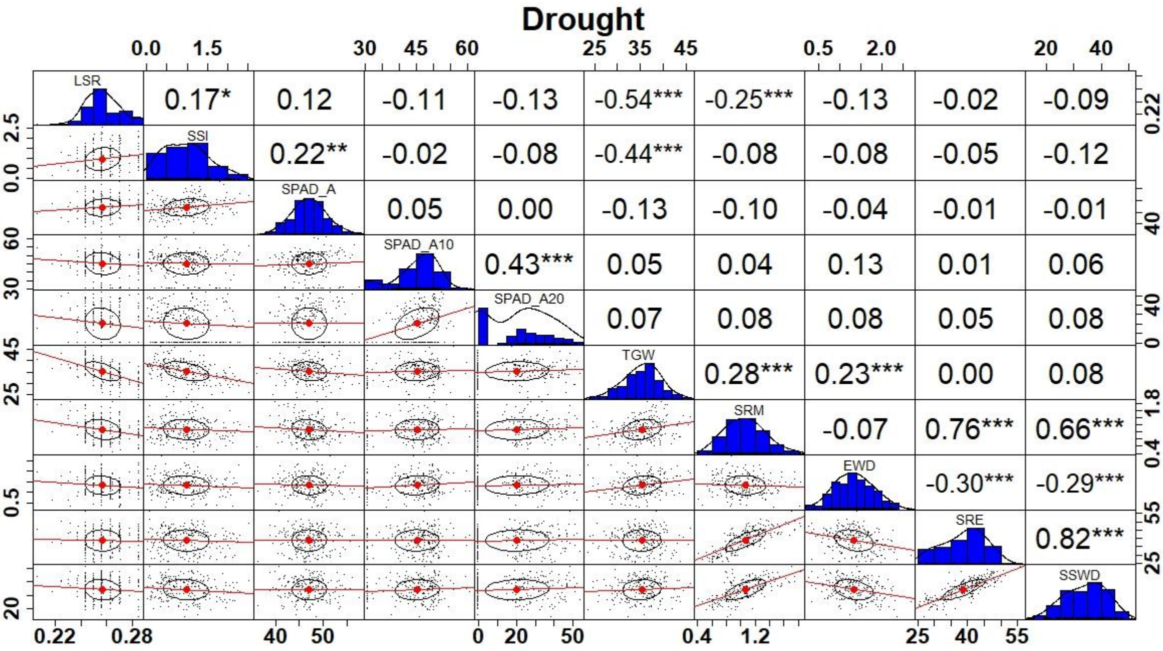


Figure 3B- Correlogram depicting correlation coefficients between traits under drought stress condition

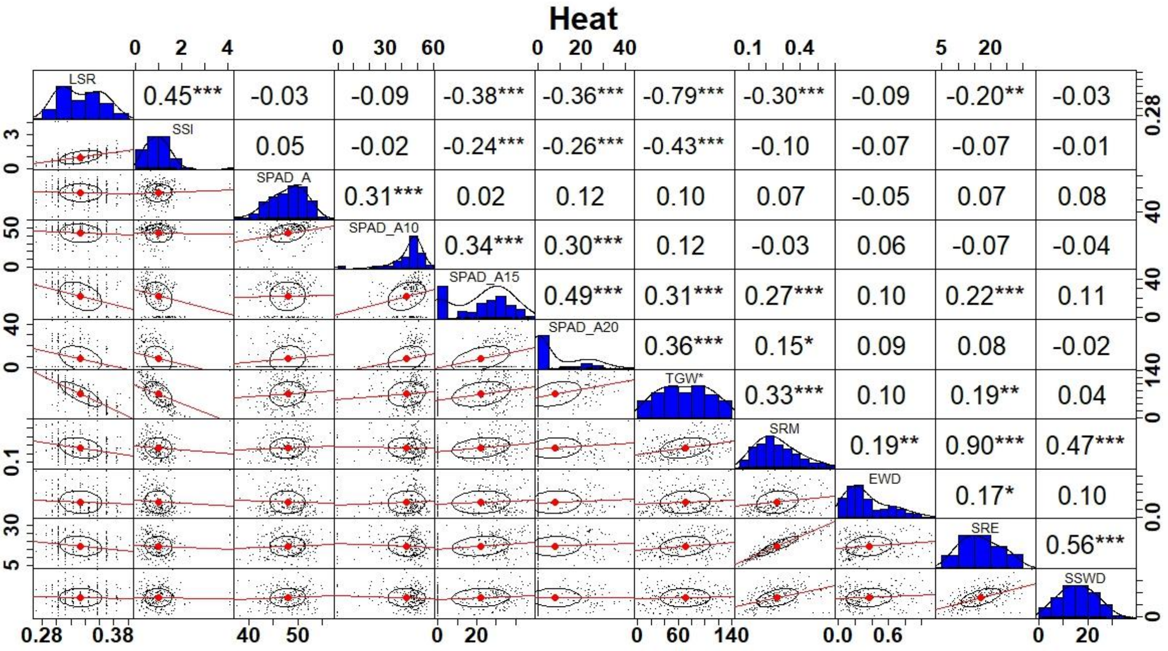


Figure 3C- Correlogram depicting correlation coefficients between traits under heat stress condition

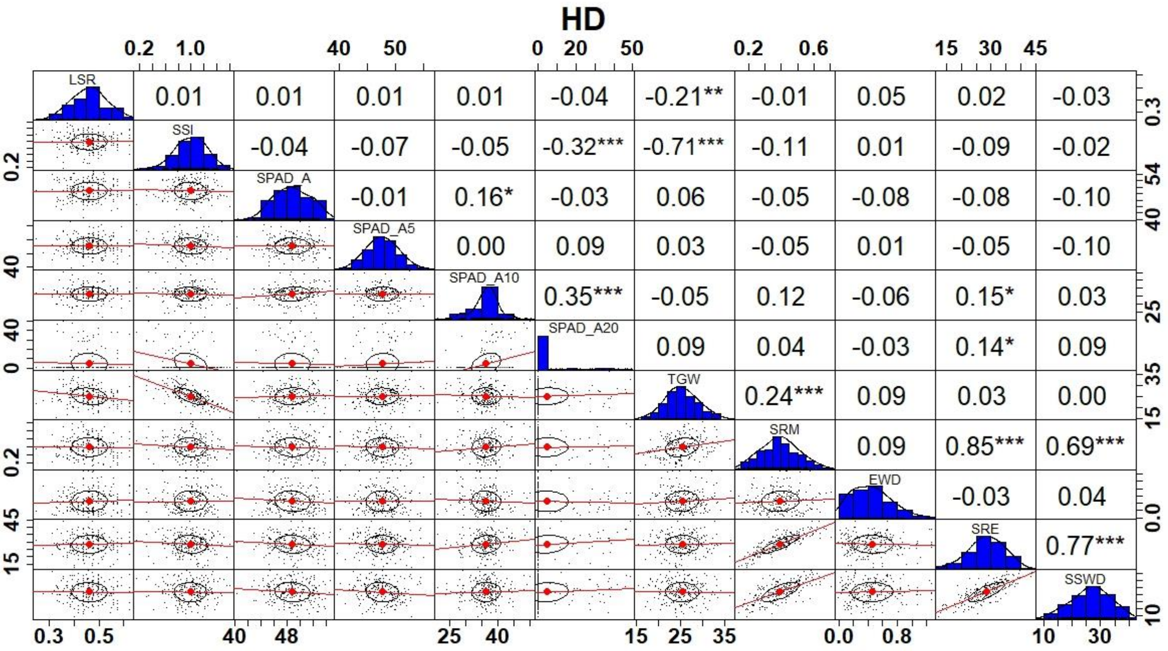


Figure 3D- Correlogram depicting correlation coefficients between traits under combined stress (HD) condition

**Control**

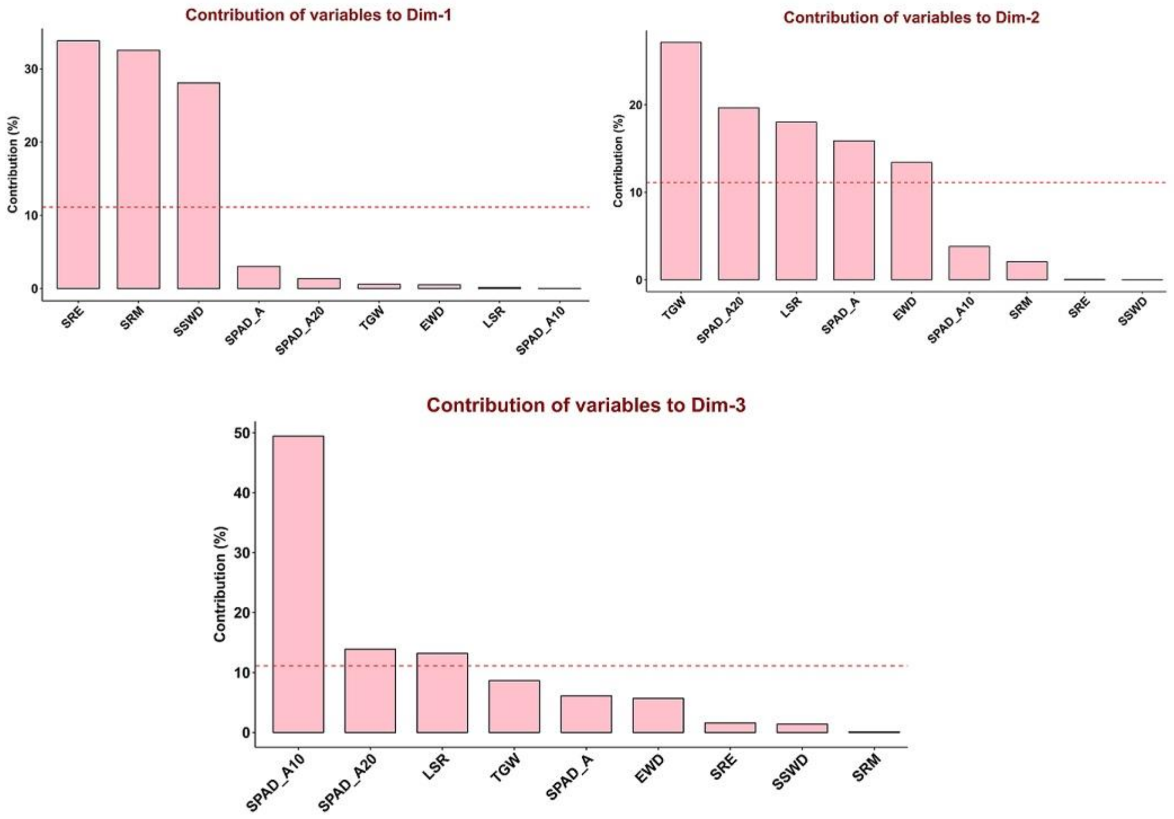


Figure 4A- Contributions of variables to PCs under control condition.

**Drought**

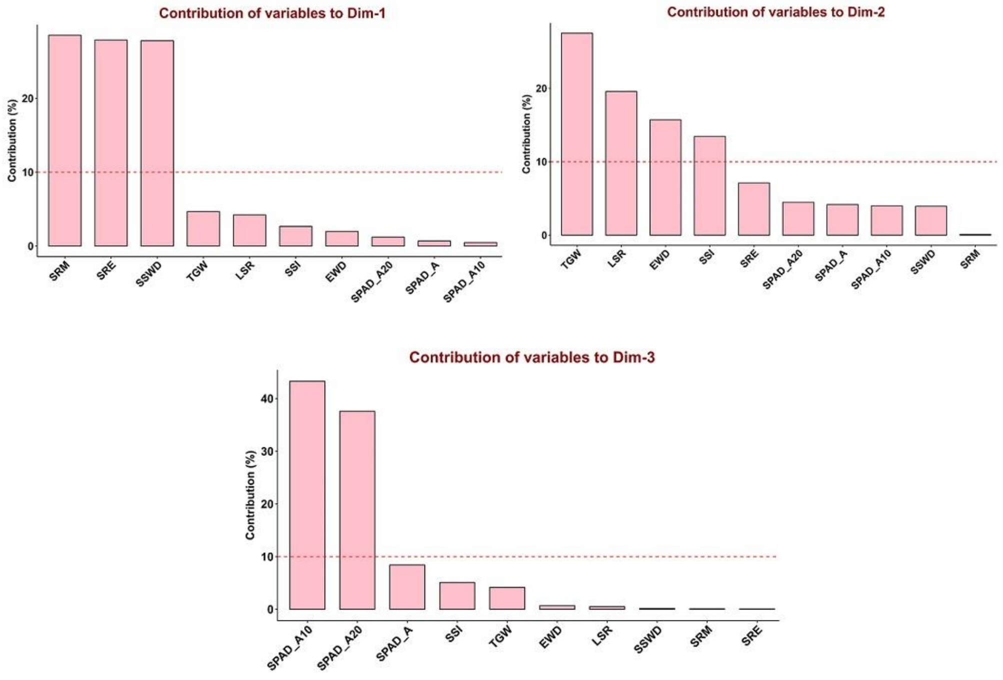


Figure 4B- Contributions of variables to PCs under drought stress condition.

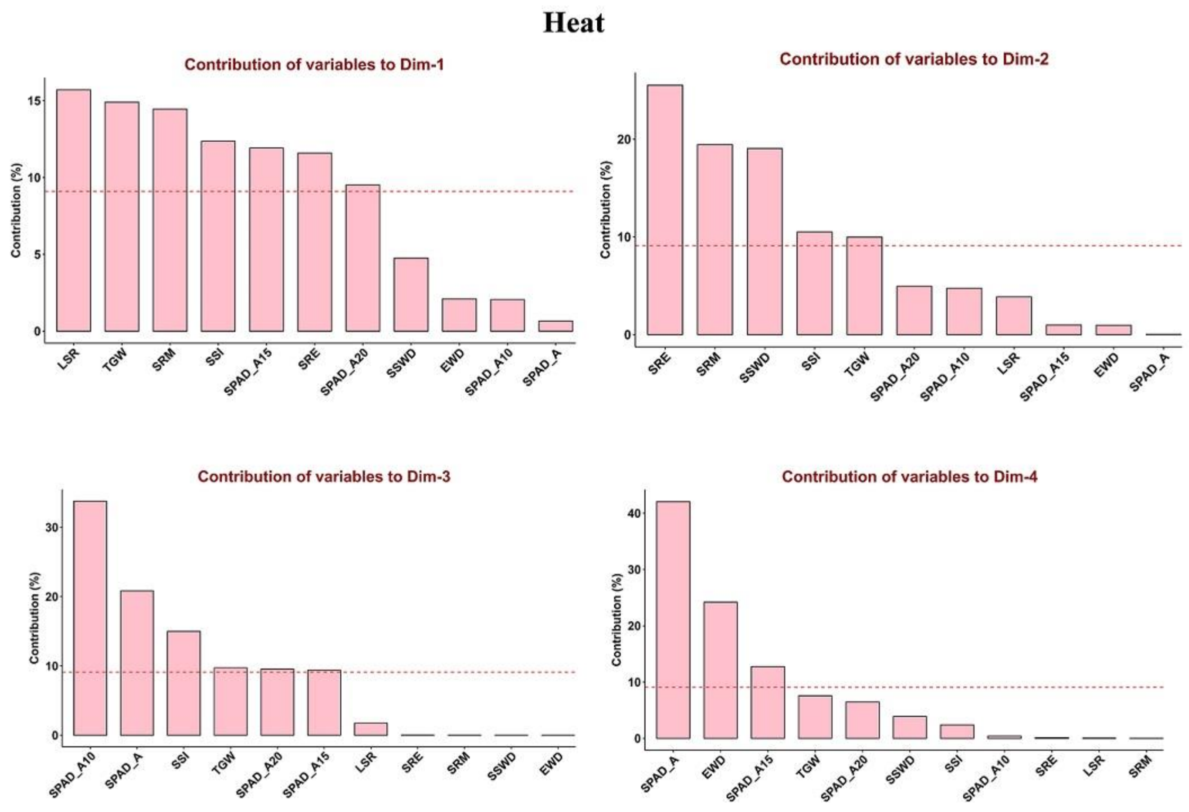


Figure 4C- Contributions of variables to PCs under heat stress condition.

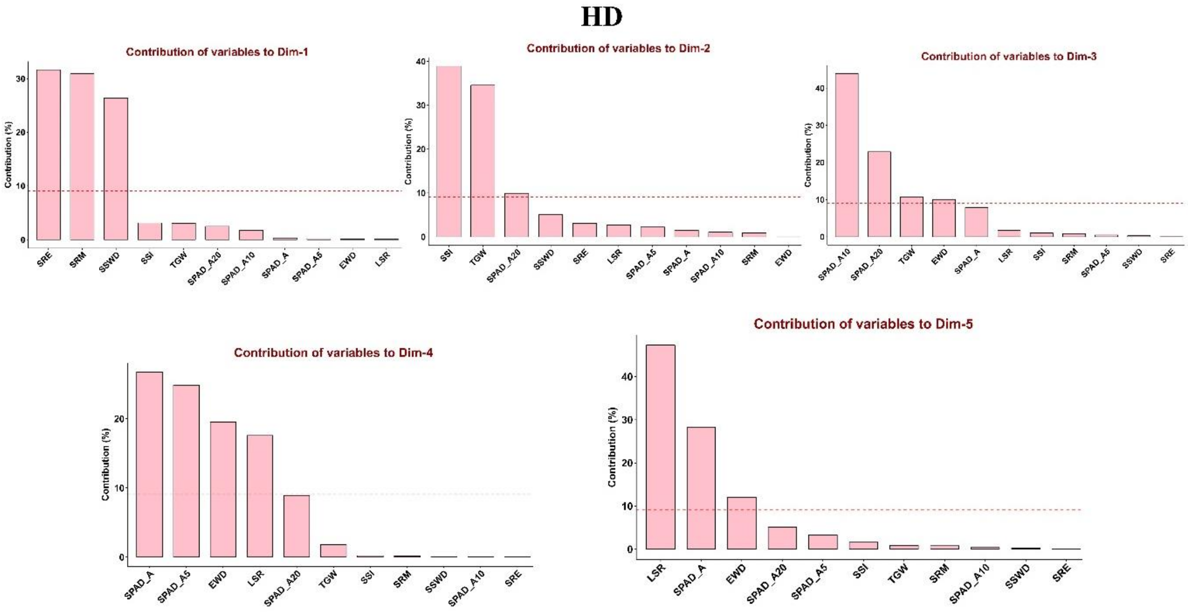


Figure 4D- Contributions of variables to PCs under combined stress (HD) condition.
